# A monolithically sculpted van der Waals nano-opto-electro-mechanical coupler

**DOI:** 10.1038/s41377-022-00734-7

**Published:** 2022-03-01

**Authors:** Tongyao Zhang, Hanwen Wang, Xiuxin Xia, Ning Yan, Xuanzhe Sha, Jinqiang Huang, Kenji Watanabe, Takashi Taniguchi, Mengjian Zhu, Lei Wang, Jiantou Gao, Xilong Liang, Chengbing Qin, Liantuan Xiao, Dongming Sun, Jing Zhang, Zheng Han, Xiaoxi Li

**Affiliations:** 1 State Key Laboratory of Quantum Optics and Quantum Optics Devices, Institute of Opto-Electronics, Shanxi University, Taiyuan, 030006 China; 2 Collaborative Innovation Center of Extreme Optics, Shanxi University, Taiyuan, 030006 China; 3 Shenyang National Laboratory for Materials Science, Institute of Metal Research, Chinese Academy of Sciences, Shenyang, 110016 China; 4 School of Material Science and Engineering, University of Science and Technology of China, Anhui, 230026 China; 5 Research Center for Functional Materials, National Institute for Materials Science, 1-1 Namiki, Tsukuba, 305-0044 Japan; 6 International Center for Materials Nanoarchitectonics, National Institute for Materials Science, 1-1 Namiki, Tsukuba, 305-0044 Japan; 7 College of Advanced Interdisciplinary Studies, National University of Defense Technology, Changsha, 410073 China; 8 The Key Laboratory of Science and Technology on Silicon Devices, Institute of Microelectronics, Chinese Academy of Sciences, Beijing, 100029 China; 9 The University of Chinese Academy of Sciences, Beijing, 100029 China; 10 State Key Laboratory of Quantum Optics and Quantum Optics Devices, Institute of Laser Spectroscopy, Shanxi University, Taiyuan, 030006 China

**Keywords:** Photonic devices, Optical properties and devices

## Abstract

The nano-opto-electro-mechanical systems (NOEMS) are a class of hybrid solid devices that hold promises in both classical and quantum manipulations of the interplay between one or more degrees of freedom in optical, electrical and mechanical modes. To date, studies of NOEMS using van der Waals (vdW) heterostructures are very limited, although vdW materials are known for emerging phenomena such as spin, valley, and topological physics. Here, we devise a universal method to easily and robustly fabricate vdW heterostructures into an architecture that hosts opto-electro-mechanical couplings in one single device. We demonstrated several functionalities, including nano-mechanical resonator, vacuum channel diodes, and ultrafast thermo-radiator, using monolithically sculpted graphene NOEMS as a platform. Optical readout of electric and magnetic field tuning of mechanical resonance in a CrOCl/graphene vdW NOEMS is further demonstrated. Our results suggest that the introduction of the vdW heterostructure into the NOEMS family will be of particular potential for the development of novel lab-on-a-chip systems.

## Introduction

Modern sensors are often designed to couple optical, electrical, and mechanical degrees of freedom in nano-scales in a single device; it thus helps in exploring many emerging properties in both classical and quantum regimes^1–5^. Systems constructed for the above purposes are defined as nano-opto-electro-mechanical system (NOEMS), which offers tremendous opportunities to control the photonic, acoustic, and electric behaviors in nanodevices, sometimes operating at very low power consumption^2^, and may be expanded in quantum systems such as superconducting circuits^1^. Recently, other than the usually adopted bulk materials, van der Waals (vdW) materials have been increasingly attractive for investigations in NOEMS. For example, a valley-mechanical coupling in a suspended monolayer MoS_2_ resonator was probed with circularly polarized lights^6^.

Indeed, two-dimensional (2D) vdW materials are of particular interest for future nano-electronic applications, owing to their peculiar mechanical and electro-magnetic performances^7–10^. More importantly, vdW layers can be vertically interfaced into arbitrary heterostructures that incorporate inter-layer coupling in themselves, giving rise to the reconstruction of band structures that are enriched of quantum and topological physics both optically and electrically^11–17^. In addition, in many circumstances, vdW functional monolayers require packaging with protecting/supporting layers such as hexagonal boron nitride (h-BN), in order to preserve their intrinsic optical properties from environmental inhomogeneities. It is thus expected that vdW heterostructures are inherently an ideal platform to serve as NOEMS. However, due to a lack of a reliable fabrication method, the NOEMS studies involving vdW heterostructures are so far very limited. Recently, a cavity-modulated photon luminescence emission behavior was reported in suspended h-BN/MoSe_2_/h-BN heterostructures^18^.

In this work, we utilize the vdW vertical assembly as a platform to devise a monolithically sculpted nano-opto-electro-mechanical coupler. By adopting the dry-transfer method^19^, we present a new fabrication process for suspending arrays of two-terminal or multi-terminal vdW heterostructures, which does not require such as critical point drier or hot acetone method to obtain suspended 2D material in conventional methods. Instead, with a one-step etching process, one can define a suspended vdW heterolayer with multifunctional potentials, which has not been achieved before. Hence, complicated vdW heterostructures with multifunctional applications could be fabricated using our new method with high sample yields. Taking the h-BN/graphene heterostructure, for example, several functionalities, including nano-mechanical resonator, vacuum channel diodes, and ultrafast thermo-radiator are realized in one single NOEMS device. Quality factors of mechanical resonances in them are found to exceed 10^3^ at room temperature. Nanovacuum channel thermionic emission diodes with on-off ratios of 10^5^ were achieved in the same nanostructure. In the meantime, the h-BN/graphene NOEMS can serve as an ultrafast thermal-radiator modulated via electrical Joule heating. The principle-of-work of the proposed monolithically sculpted nano-opto-electro-mechanical coupler can be expanded to a wide variety of 2D materials and their heterostructures, which sheds light on future lab-on-a-chip electronic systems based on vdW NOEMS.

## Results

### Monolithically sculpted vdW heterostructure mechanical resonator

**Fig. 1:**
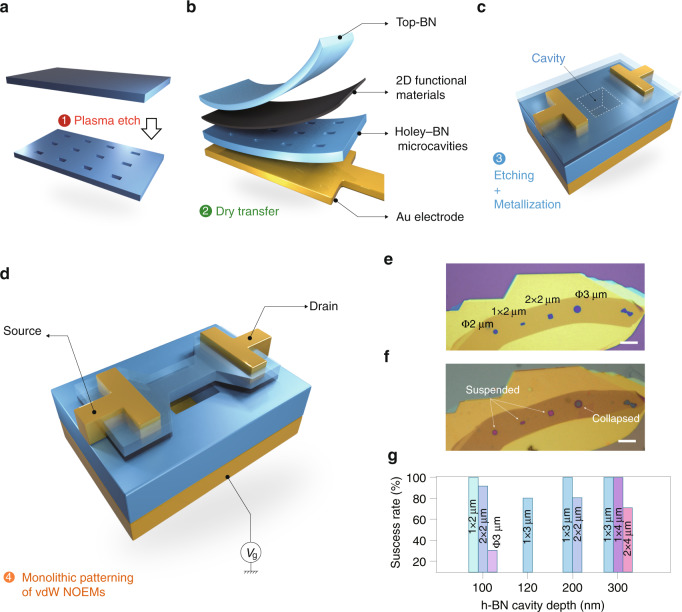
Monolithically sculpted vdW heterostructure NOEMS. **a**–**d** Art view of the workflow for patterning suspended vdW heterostructure beams on h-BN cavities, with 2D functional materials involved, thus forming a NOEMS structure. **e**–**f** A typical test on the suspension of vdW heterostructure on pre-patterned h-BN holes, with a global top vdW layer (**f**) deposited onto (**e**). **g** A statistic on the success rate of suspension (at step 3 in Fig. 1) of h-BN/graphene heterostructure on h-BN cavities with different h-BN cavity depths. The different colors denote lateral sizes of the suspended areas. Ten samples were fabricated for each size for the statistic in (**g**)

The advantages of the NOEMS made of suspended vdW heterostructures in this work are that vdW materials exhibit enriched spin, valley, and topological properties, with usually ~10^2^ MHz resonance frequency that can be further coupled to an energy scale of such as Landau levels^20^. Furthermore, the conduction channel of graphene allows us to utilize the suspended nanostructure as a filament to demonstrate ultrafast electron emitter, which is of stronger mechanical strength and can be patterned into multi-terminal configuration, which was not achievable in those devices constructed out of a single atomic layer studied before^21–23^. Moreover, vdW based devices are believed to be resistant under radiation environment, as will be discussed in the next sections. Now we introduce the example of application of mechanical resonator realized using the method in this work. Holey h-BN with thicknesses of about 100–300 nm were first prepared with plasma etching, as shown in Fig. 1a. Multi-layered vdW heterostructures are then deposited onto the holey h-BN (Fig. 1b), forming vdW films sealed cavities shown in Fig. 1c. Micrometer-sized suspended vdW multi-layered beams can thus be fabricated by a sole final step of dry etching. As shown in Fig. 1d, the heterostructure suspended beams on pre-patterned cavities can then serve as nano-mechanical resonators with 2D materials functional layers ready to be coupled for optical and electrical measurements. It is found that with a cavity depth of about 200 nm, the success rate of suspension is 100% when the lateral sizes are less than 3 μm (Fig. 1e, f), which is quite robust and facile as compared to the conventional monolayered suspension of 2D materials, while the latter usually adopts dedicated process using critical point drier or a hot acetone technique^24^. More details of the workflow of the fabrication process can be found in Supplementary Fig. S1.

**Fig. 2:**
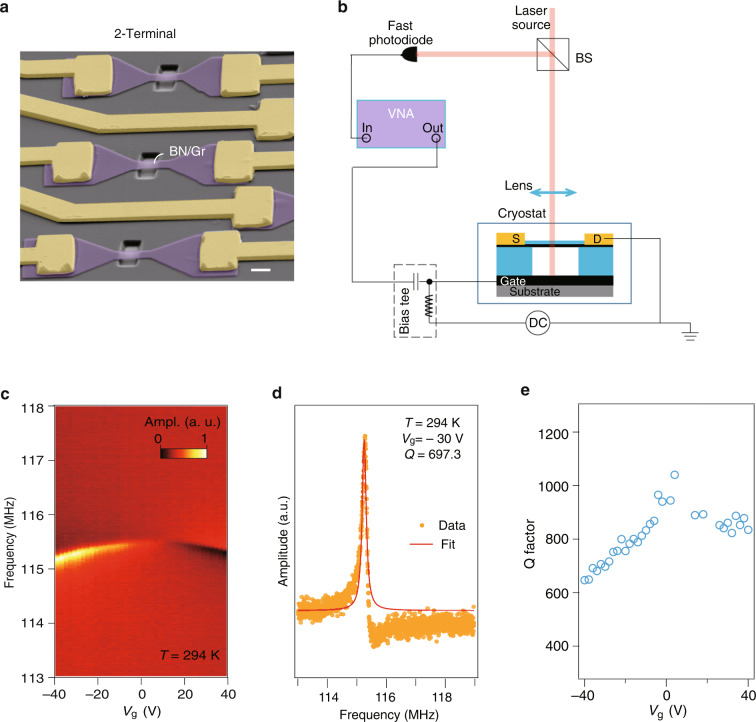
Nano-mechanical resonances in vdW heterostructure NOEMS. **a** False colored SEM image of a typical resonator using vdW bilayer h-BN/graphene as the suspension channel. **b** Schematic of the setup for optical probe of the mechanical resonance. **c** Color map of the resonance frequency as a function of gate voltage measured in a typical vdW NOEMS. The amplitude data with positive and negative values are mapped to a renormalized range of [0,1]. **d** Line profile of the resonance peak at *V*_g_ = –30 V, with a Lorentzian fit in the red solid curve. **e** Q factors obtained from (**c**)

Figure 2a illustrates typical devices of two-terminal vdW heterostructure (h-BN/graphene as an example was demonstrated here) resonators fabricated using the method in Fig. 1. To determine the mechanical resonance of the vdW NOEMS, the suspended h-BN/graphene beams were modulated by an AC voltage capacitively coupled with the out-of-plane motion. As shown in Fig. 2b, an optical interferometry setup was established to sensitively monitor the displacement of the emitters and a fast photodiode was used to detect the interferometric strength of the reflected laser. A vector network analyzer (VNA) was applied as an AC excitation to actuate the resonators and to read the mechanical resonance. DC back gate voltage was provided by a separate voltage source. By testing the h-BN/graphene suspended heterostructure, we obtained typical gate-tunable resonance amplitude versus AC-driven frequency and DC gate voltage at room temperature, with resonance frequency *f*_0_ of 115–116 MHz, as shown in Fig. 2c. A line cut at *V*_g_ = −30 V is illustrated in Fig. 2d, in which the resonance peak is fitted using a single Lorentzian, yielding a quality factor Q of 697.3. Figure 2e shows the extracted Q factors as a function of gate voltage in the same device. Measurements of resonance at lower temperature and control samples are shown in Supplementary Fig. S2.

### vdW vertical vacuum channel thermionic emission diodes

**Fig. 3:**
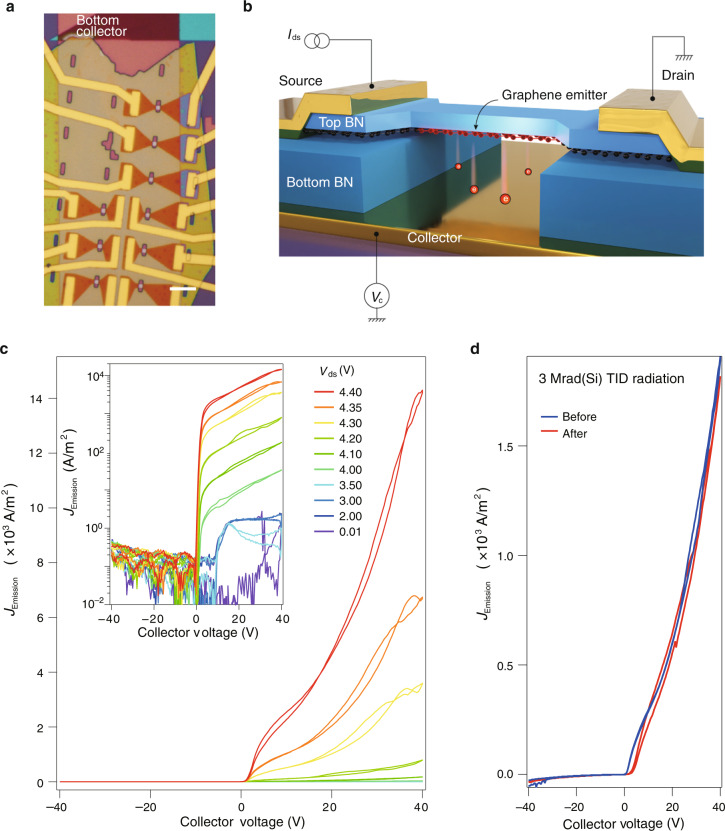
Thermionic emission diode behavior in h-BN/graphene NOEMS. **a** Optical micrograph image of the arrays of nanosized vdW NOEMS. Scale bar = 5 μm. **b** Art view of the architecture of the “vintage” vacuum diode made of vdW heterostructure. **c** Thermionic emission current density *J*_Emission_–*V*_Collector_ curves in a *V*_Collector_ range of ±40 V recorded in a typical device. The same data are plotted in a log scale in the inset. Traces and retraces are recorded. Data were obtained at 300 K in a vacuum chamber of about 10^−2^ mbar. **d** TID radiation effects characterization of the emission current in a typical vdW thermionic emission diode device shown in (**a**)

In the following, we demonstrate that the fabricated vdW NOEMS can be functioning as vertical vacuum channel thermionic emission diodes. Optical image of such solid device of vacuum channeled diodes and an art view of the finish of the device architecture are shown in Fig. 3a, b, respectively. In this configuration, the holey h-BN is used as a supporting dielectric with the deep holes serving as vacuum channels. In consequence, when exerted with a large enough current in the emitter (suspended graphene channel that is supported by a top h-BN beam), graphene will become a filament that shines a bright light. And thermionic electron emission can take place when a certain positive collector (both Au and graphite can be used as the collector, as shown in Supplementary Fig. S3) voltage is applied.

For monolayer graphene vdW vertical thermionic emission diode, it is rather vulnerable to the thermionic electron emission tests. Many of them cannot survive more than two cycles of emission, with the emitter channels collapsing easily, as shown in Supplementary Figs. S4 and S5. In the following, we will mainly focus on few-layered graphene emitters. Typical rectifying curves can be seen in the vertical configuration with few-layered graphene emitters, shown in the linear and log scale in Fig. 3c and its inset, respectively. And the thermionic emission onset voltage is about 3–4 V, with the maximum emission current (before the emitter channel is burnt down) at the order of 10 nA. Subthreshold swing (SS) is extracted to be at the order of 200 mV/decade, comparable with the values obtained in previously reported nanosized vacuum tubes^25,26^. For thermionic emission diodes, we define the ratio of maximum emission current to minimum emission current versus collector voltage at a certain *V*_ds_ as an on-off ratio. According to the inset shown in Fig. 3c, the on-off ratios can reach a level up to 10^5^, in the condition of *V*_ds_ = 4.4 V.

A simplified Richardson–Dushman model depicts that the electrons emitted during the process of thermionic emission depend upon the surface area of the metal surface and the temperature of the surface, written as ${I}_{\mathrm{Emission}} = {AT}^{2}\mathrm{exp} {(- {WK}^{-1}{T}^{-1})}$^27^. Here, *A* is a constant that is proportional to the emitter surface and being materials-dependent, while *W* and *T* are work function and temperature of the emitter material, respectively. By assuming that the thermionic emission follows the law of the blackbody radiation, we measured the spectra at room temperature in a homemade vacuum chamber with the setup illustrated in Supplementary Figs. S6 and S7. The corresponding temperature can be determined by the Planck formula (Supplementary Figs. S8–S12). By plotting the value of *I*_Emission_ as a function of *T*^2^exp(−*WK*^−1^*T*^−1^) with the work function of 4.5 eV, we yield an *A* ~14.8 Acm^−2^K^−2^, qualitative agreement with the experimental observations in other metallic materials^28^. However, more dedicated modeling may be needed to quantitatively understand the exact behaviors in the studied devices, as the Richardson model may not be sufficient when it comes to low dimensional systems^29^.

We also performed the total ionizing dose (TID) effect experiment in the vdW NOEMS, which is important for our device to be used in radiation environments. In general, TID effect is treated as a long-term cumulative radiation effect. The ionized charges induced by high-energy rays and particles are trapped at either the insulators interfaces or in the bulk region that can cause turn-on voltage shift and leakage current increase. The TID irradiation experiments were performed in a ^60^Co gamma rays source with a dose rate of 50 rad(Si)s^–1^. During the irradiation, the vdW vertical thermionic emission diodes were established in a float state. As shown in Fig. 3d, the thermionic electron emission behaviors before and after the radiation with a TID dose of 3 Mrad(Si) are almost the same, exhibiting great stabilities in terms of TID effect similar to other nanosized vacuum channel transistors^30^. We also compare the characteristics of our vdW thermionic emission diodes with other nanosized thermionic emission devices. As shown in Supplementary Table S1, characteristic parameters such as On/Off emission ratio, collector voltage, and SS are summarized. It is seen that the vdW thermionic emission diodes investigated in this work show maximum On/Off emission ratio of about 10^5^, and a SS reaching 200 mV/decade. Furthermore, the devices reported in our work are integratable using the vdW stacking technique, which is fully compatible with the solid-state device fabrication process.

### Ultrafast thermal-radiator realized in vdW NOEMS

We now take the h-BN/graphene NOEMS as an example to illustrate the functionality as an ultrafast thermal-radiator. A rectangular waveform of AC current was exerted into the suspended h-BN/graphene channel, with a width of the peak of about 10 ns and a DC biased to tune periodically the Joule heating. Hence pulses of blackbody radiation can be detected via a time-resolved single-photon detector.

**Fig. 4:**
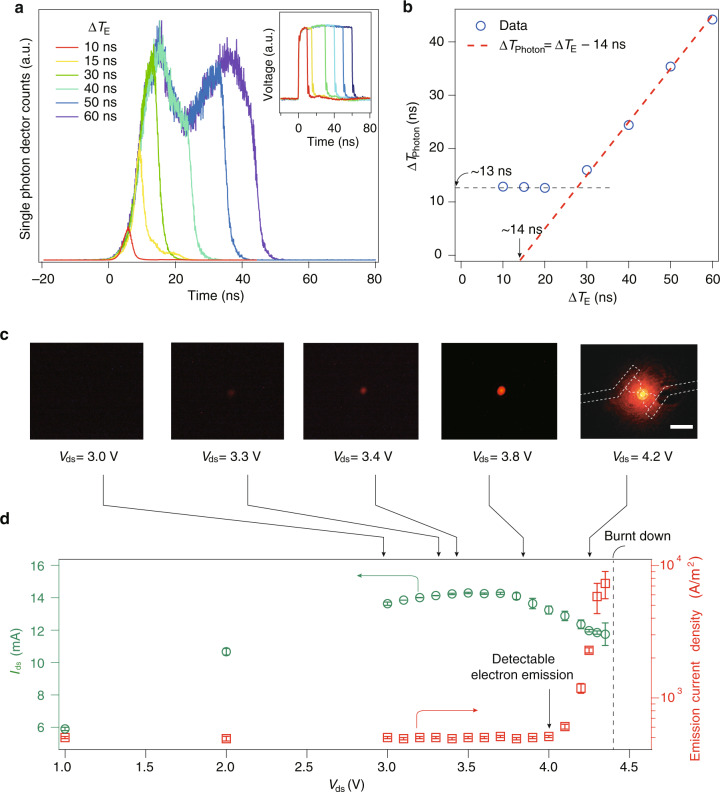
Dynamics of the vdW NOEMS functioning as ultrafast thermo-radiators. **a** Typical ultrafast blackbody radiation excited by square electrical pulse sequences with a fixed repetition rate of 100 kHz (*T* = 10 μs) but various electrical pulse duration Δ*T*_E_ = 10, 15, 30, 40, 50, and 60 ns, respectively. Inset presents the input voltage signals (electrical voltage pulses of the excitation trace in ns time scale recorded by an oscilloscope) on the tested devices. **b** Electrical pulse width Δ*T*_E_ versus the corresponding light-emission pulse width Δ*T*_Photon_ extracted from (**a**). Dashed lines are linear fits. **c** Optical image captured in the CCD camera at fixed *V*_ds_, showing the status of light emission of the vertical vdW thermionic emission diode. White dashed lines illustrate the top view profile of the emitter. Scale bar = 5 μm. Data were obtained at 300 K and under a vacuum condition of about 10^−2^ mbar. A collector voltage of +40 V was maintained throughout the measurements. **d** Profiles of thermionic electron emission *I*_Emission_ (red squares) and *I*_ds_ (green circles) as a function of *V*_ds_. Error bars represent the fluctuation of currents recorded during measurements

Figure 4a shows typical ultrafast blackbody radiation excited by square electrical pulse sequences with a fixed repetition rate of 100 kHz (*T* = 10 μs) but various electrical pulse duration Δ*T*_E_ = 10, 15, 30, 40, 50, and 60 ns, respectively. The input voltage signals (electrical voltage pulses of the excitation trace in ns time scale recorded by an oscilloscope) on the tested devices are presented in the inset in Fig. 4a. And ultrafast blackbody radiation in response to AC electrical current injection at different repetition frequencies is shown in Supplementary Fig. S13. It is noticed that, during the test, a bias voltage of *V*_dc_ = 0.8 V plus an AC voltage of around 2.1 V is applied. Electrical pulse width Δ*T*_E_ versus the corresponding full width at half maximum Δ*T*_Photon_ of the light-emission pulse were extracted from Fig. 4a. As shown in Fig. 4b, a quasi-linear relationship with a slope of the unity between Δ*T*_Photon_ and Δ*T*_E_ is found for Δ*T*_E_ > 30 ns. An intercept of ~14 ns on the Δ*T*_E_ axis can be seen, which is attributed to a sum of the rise time *T*_rise_ plus the fall time *T*_fall_. Moreover, in the range of Δ*T*_E_ < 20 ns, Δ*T*_Photon_ levels off at ~13 ns (black dashed line). This value is in agreement with that of *T*_rise_ + *T*_fall_. Assuming the *T*_rise_ and *T*_fall_ are the same; therefore, a cooling time of ~7 ns of the nanovacuum channel thermionic emission diode can be estimated in the condition of a sufficient long repetition period (~10 μs). The above dynamic analysis thus provides insights into such nanosized suspended thermionic emission systems.

Figure 4c, d show the static Joule heating regime of the h-BN/graphene NOEMS. Static thermionic emission current *I*_Emission_ as well as emitter channel current *I*_ds_ are recorded, in Fig. 4d. It is seen that the *I*–*V* curve of the emitter channel (green circles in Fig. 4d) exhibits a saturation behavior in *I*_ds_ above *V*_ds_ ~3.5 V. It is noteworthy that, after the saturation regime of *I*_ds_ (3 V < *V*_ds_ < 3.7 V), a clear drop of *I*_ds_ is seen when the *V*_ds_ is further increased. We define this point as the onset of detectable thermionic emission current, as indicated by the black arrow in Fig. 4d. At *V*_ds_ = 4.0 V, emission current (red squared in Fig. 4d) can be captured from the collector electrode, which rockets into 10 nA at *V*_ds_ = 4.2 V, and breaks down at 4.3 V. Similar behavior is seen in multiple samples. In this setup, a Keithley 2400 multi-meter was used to detect emission current, and the sub-1 nA emission may be overlooked. Corresponding visible light emissions are also given in Fig. 4c for each stage of *V*_ds_.

## Discussion

To this stage, we have demonstrated a multifunctional NOEMS fabricated by monolithically sculpting a vdW heterostructure. Taking h-BN/graphene bilayer system as an example, optically, it can serve as an ultrafast thermal-radiator with cavity resonant peak tunable by the depth of the cavity (Supplementary Fig. S9). Electrically, the system can be regarded as a nano-version of the “vintage” thermionic emission diode with a vertical vacuum channel and solid-state device structure. In addition, mechanically the fabricated system can well play the role of a mechanical resonator with Q factors reaching 10^3^ at room temperature. The observed multi-functionalities in a single solid device well define a prototype of NOEMS using vdW heterostructure as a platform.

Despite the demonstrated versatility of the vdW NOEMS in this work, there are yet rooms in them to improve the performances such as thermionic emission efficiency (i.e., to decrease the total power consumption) and the emission currents, as compared to those Si-based nanosized vacuum channel vacuum electron cold emitters^21,22,30–37^. For example, to enhance the thermionic electron emission current, surface coating of oxides on the graphene emitter to further lower its work function may be our future studies^38^.

As shown in Supplementary Fig. S14a, thanks to the enriched library of 2D materials, the current studied system can thus be expanded into those of electronic and optical properties that involve such as spin and valley degrees of freedom (Supplementary Fig. S14b, c). Moreover, it is noticed that the unique fabrication process of the vdW NOEMS also allows the realizations of such as multiple-terminal suspended vdW conduction channels, shown in Supplementary Fig. S14d, which is of potential for opto-electro-mechanical studies especially in the quantum Hall regime, which was considered technically extremely difficult before^20,39^.

To further demonstrate the interplay of optical, electrical, and mechanical degrees of freedom in one single NOEMS using our technique, we now discuss a platform for optical readout of electric and magnetic field tuning of mechanical resonance in a CrOCl/graphene vdW NOEMS. Recently, the temperature dependence of mechanical resonance in magnetic semiconductors, such as XPS_3_ (X = Fe, Mn, Ni)^40^ and Cr_2_Ge_2_Te_6_^41^, are investigated. Meanwhile, magnetic field-driven redshift of mechanical resonance in the anti-ferromagnetic (AF) vdW insulators from AF to ferromagnetic transition in CrI_3_^42^. Here, we adopt an AF insulator CrOCl, which is known to exhibit enriched magnetic phase transitions^43,44^.

**Fig. 5:**
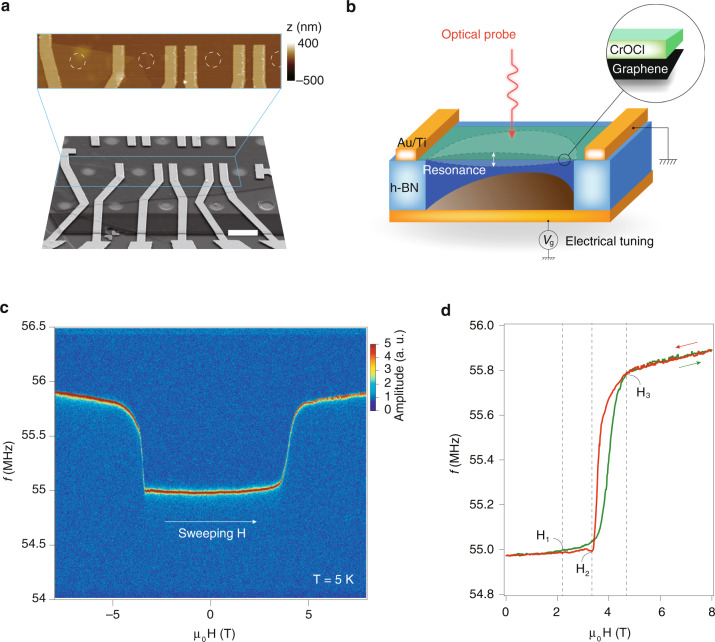
Demonstration of a CrOCl/graphene vdW NOEMS using our technique. **a** Bird view of SEM image of the CrOCl/graphene NOEMS array using the method described in Fig. 1, with the boxed region scanned by AFM. It is seen that the drum-like cavities (dashed circles in the AFM image) are invisible under AFM. Scale bar = 5 μm. **b** A cartoon illustration of the as-prepared of CrOCl/graphene NOEMS. **c** Color map showing the resonance frequency of the CrOCl/graphene NOEMS in the parameter space of frequency and magnetic fields at a temperature of 5 K. **d** Line profile of the resonance frequency *f*_0_ extracted from (**c**), as a function of magnetic field. Data were obtained in trace and retrace, with a magnetic phase transition from H_1_ to H_3_, with *f*_0_ blueshifts of about 0.8 MHz

As shown in the SEM and AFM images in Fig. 5a, using the fabrication technique described in Fig. 1, the CrOCl/graphene heterostructure are fabricated into arrays of drum-like resonators, with the monolayer graphene acting as the capacitive coupling layer to the AC and DC gate voltages, and the 10 nm CrOCl layer is the AF layer in this NOEMS. A schematic picture of the coupling between optical probe, mechanical resonance, and electrical tuning is given in Fig. 5b. At the base temperature of 5 K in our setup, the resonant frequency *f*_0_ at the ground state is measured by the optical interferometer to be ~55 MHz, and at about 4 T, a shift of ~0.8 MHz is observed, an order of magnitude higher than those reported in other AF vdW resonators^42^. Gate tuning of *f*_0_, and more details of the temperature dependences can be found in Supplementary Figs. S15–S18. Notice that the blueshift of *f*_0_ corresponds to an increase of strain in the membrane, which is caused by the magnetostriction effect in the few-layered CrOCl. By examining the trace and retrace of *f*_0_-*μ*_0_*H* curve in Fig. 5d, one can see that the system undergoes three magnetic phase transitions (at H_1_, H_2_, and H_3_, respectively), which is in agreement with the report elsewhere^43,44^. The CrOCl crystal has a monoclinic phase below the Néel temperature, and exhibits a so-called stripy AF-↑↑↓↓ magnetic ground state due to magnetoelastic coupling^44^. It then reaches a ferrimagnetic phase ↑↑↑↓↓ above H_3_. It is thus inferred that the few-layered CrOCl may have a structural phase transition above H_3_, with the lattice constant shrunk and hence a stiffness enhancement in the membrane. Our technique thus provides a NOEMS platform for opto-mechanical detection of complex electro-magneto responses in vdW heterostructures.

To conclude, we devised a monolithically sculpted nano-opto-electro-mechanical coupler with high sample yields. Multi-functionalities, including mechanical, optical, and electrical operations, are integrated into one single vdW NOEMS device made of h-BN/graphene. For example, it can serve as an ultrafast thermal-radiator, a nano-version of the “vintage” thermionic emission diode, and a mechanical resonator. In principle, the proposed monolithically sculpted nano-opto-electro-mechanical vdW heterostructure system can be expanded to a wide variety of 2D materials, and can also be shaped into multi-terminal NOEMS. Optical readout of electric and magnetic field tuning of mechanical resonance in a CrOCl/graphene vdW NOEMS is further demonstrated. Our findings suggest that, as shown in Supplementary Fig. S14, the monolithically sculpted suspended vdW assemblies proposed here opens up opportunities for future NOEMS studies with both classical and quantum degrees of freedom, including spin- and valley-tronics, as well as mechanical coupling to possible quantum opto-electronic states.

## Materials and methods

The BN-encapsulated graphene was fabricated in an ambient condition, using the dry-transfer method. A Bruker Dimension Icon AFM was used for thicknesses and morphology tests. Electron beam lithography was done using a Zeiss Sigma 300 SEM with an Raith Elphy Quantum graphic writer. The high precision of current measurements of the devices was measured using a LakeShore vacuum probe station at room temperature, with an Agilent B1500A Semiconductor Device Parameter Analyzer.

A homemade vacuum chamber (4 cm × 5 cm × 2 cm in size) is used for monitoring the electrical and optical performances of the vdW heterostructure NOEMS simultaneously under a vacuum of about 10^−2^ mbar (Supplementary Figs. S6 and S7). The vacuum test chamber was inversely mounted on an X–Y scanning stage on top of a microscope for optical measurements, while the electrical wiring is connected from a standard chip carrier via a vacuum feed-through. To locate the graphene emitter, we first find the sample by optical microscope. Then a mild voltage of *V*_ds_ ~3 V (corresponds to an *I*_ds_ of ~10 mA) is applied onto the emitter channel, in which condition no observable light emission can be seen by the CCD camera. However, at such current density, a very faint blackbody radiation starts to occur, which can be captured by the single-photon detector. One can thus carry out spatial mapping and precisely locate the center position of the emitter (Supplementary Fig. S8).

For optical interferometric detection, the beam of a temperature-controlled semiconductor laser (λ = 780 nm) was focused on vdW NOEMS samples with a spot radius of ~2 μm and its power density was kept in a range from 4 to 8 μWμm^−2^. The samples were mounted in a helium-free cryostat under a vacuum below 10^−2^ mbar. The reflected laser was detected by a fast photoreceiver with a –3 dB bandwidth at 650 MHz. The actuation AC voltage (lower than ~4 mV) between the graphene emitter and the back gate was supplied by a VNA to modulate the reflection of the optical cavity formed by the graphene emitter and the collector. The out-of-plane displacement was monitored by such optical interferometry and measured as a function of driven frequency by the same VNA. Mechanical resonance data of different h-BN/graphene samples at low temperatures are shown in Supplementary Fig. S2.

## Supplementary information

Supplementary Material

## Data Availability

The data that support the findings of this study are available at Zenodo, 10.5281/zenodo.4725515.

## Code availability

The computational codes that support the findings of this study are available from the corresponding authors upon reasonable request.
