# Supplementary material for: A monolithically sculpted van der Waals nano-opto-electro-mechanical coupler
Source: Light Sci Appl. 2022 Mar 1;11:48. doi: 10.1038/s41377-022-00734-7 (PMC8888553; doi:10.1038/s41377-022-00734-7)
Supplement: Supplementary file 1 — Supplementary Material [file 41377_2022_734_MOESM1_ESM.doc]

Supplementary Materials for

**A monolithically sculpted van der Waals nano-opto-electro-mechanical coupler**

Zhang, Wang, Xia, Yan, *et al.*


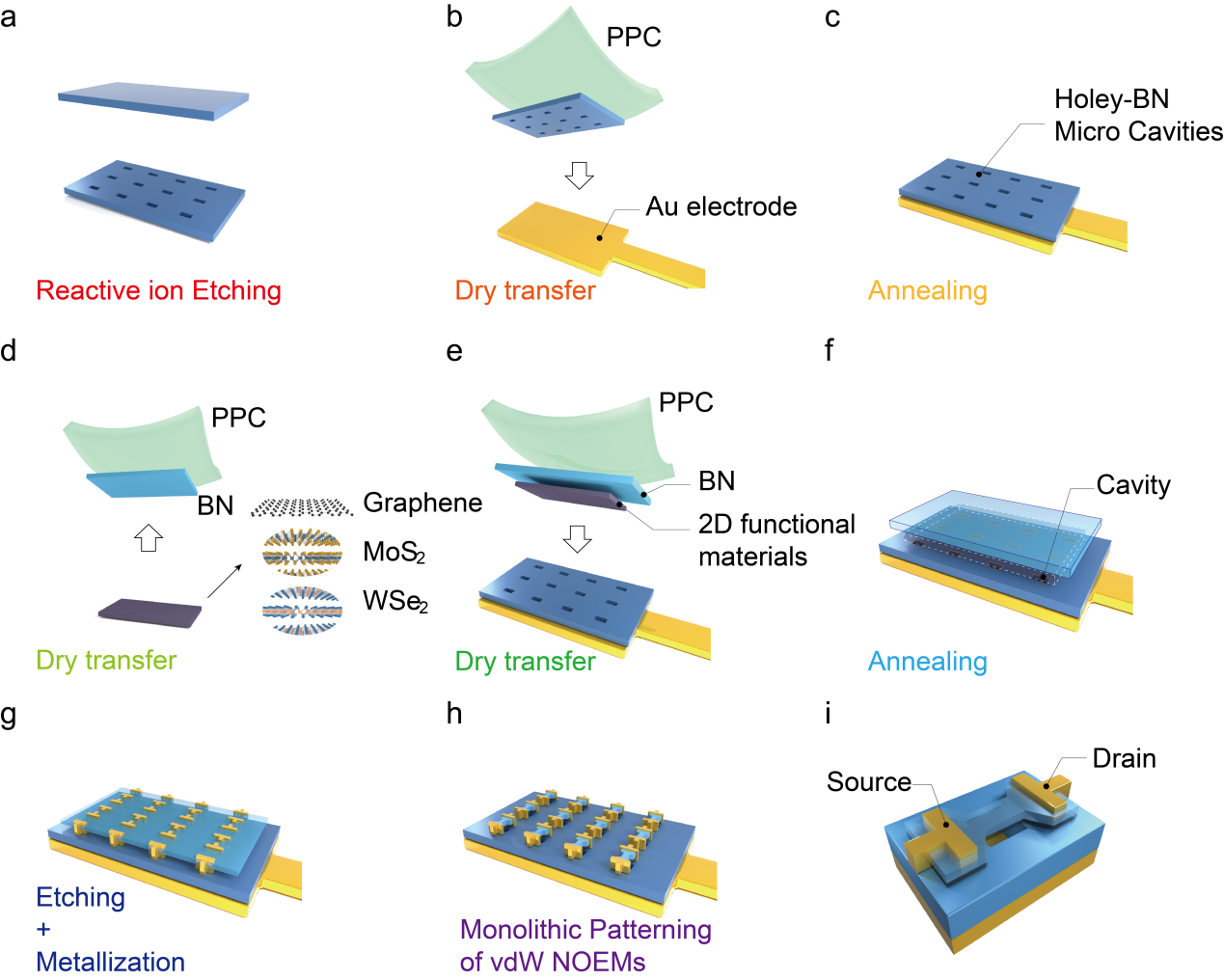


**Figure S1. Fabrication process of the monolithically sculpted vdW nano-opto-electro-mechanical systems (NOEMS) couplers.** (a) The process of holes (1 μm×3 μm in size, for example) patterning in the spacer h-BN (200-400 nm in thickness) by reactive ion etching (RIE). (b) Transferring the holed h-BN onto the surface of Au (or graphite) modulating electrode by PPC (Propylene-Carbonate). (c) Completely removing the residual PPC by vacuum annealing at 350 ℃ for 60 min. (d)-(f) Transferring the h-BN/2D functional materials (Graphene, MoS_2_, WSe_2_, and etc.) stacks onto the surface of the holed h-BN/Au stack using a dry transfer method, and annealing at 350 ℃ for 60 min. (g) Cr/Au (5 nm/200 nm) electrodes are deposited using thermal evaporation in the etched trenches followed by standard electron beam lithography (EBL). (h) Another RIE process to pattern the emitter. Electron beam resists are removed by solvent. (i) Enlarge view of the NOEMS coupler in (h). This process gives over 90% sample yield, which is very reliable and without the requirement of the critical point drying procedure.


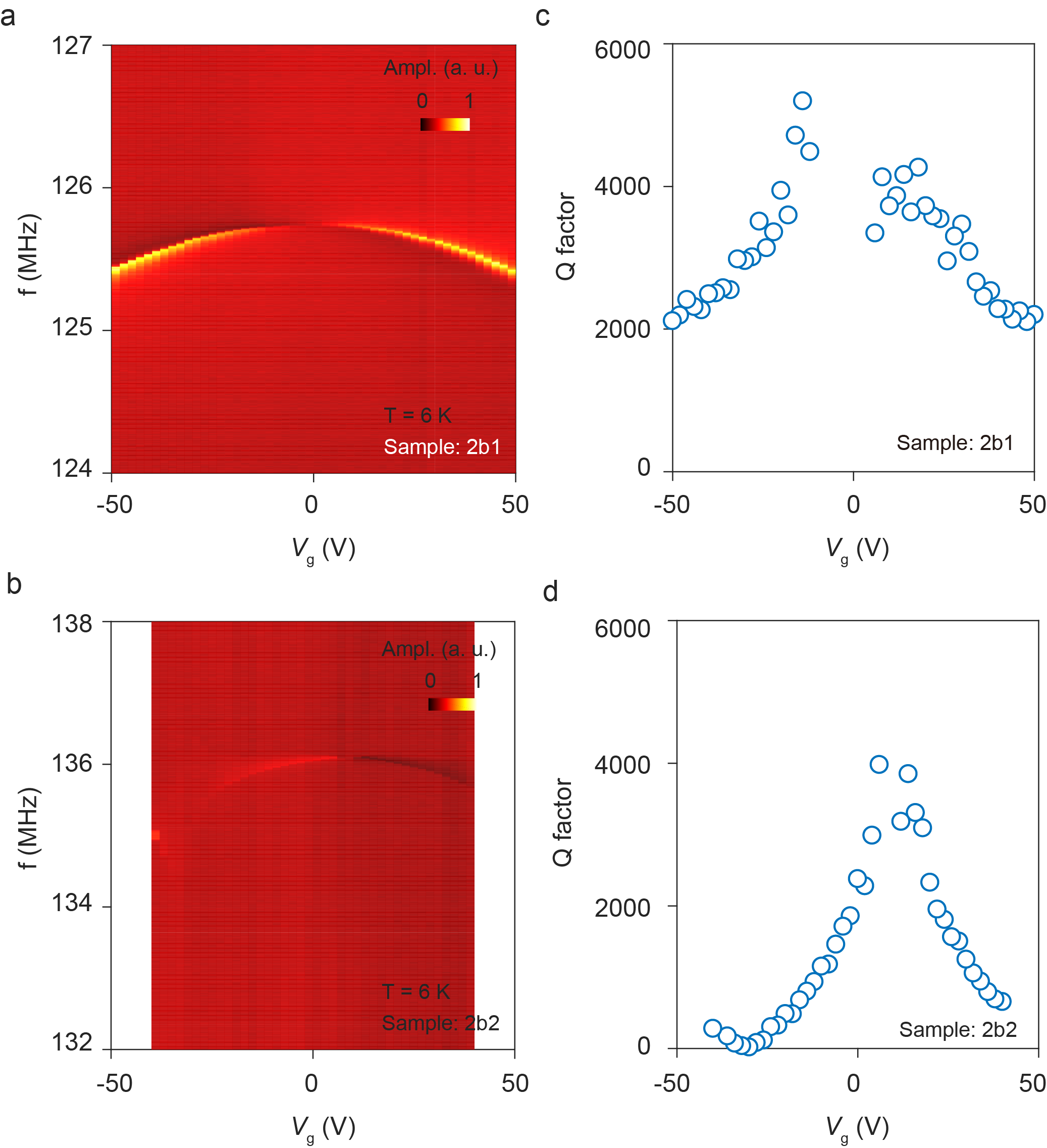


**Figure S2. Optical interferometry and mechanical resonance at low temperatures.** (a)-(b) 2D mapping of resonance amplitude versus driven frequency and DC *V*_g_ from two different samples respectively. Data obtained at 6 K. (c)-(d) Q factors versus *V*_g_ calculated from (c) and (d) respectively. The quality factor of van der Waals vacuum tubes increased from < 1000 at room temperature (Fig. 2 in the main text) to > 4000 at low temperature (Fig. S2 and Fig. S15). It is seen that, when acting like nano-mechanical resonators, the vdW NOEMS exhibits comparable Q-factors as compared to previously reported state-of-art works.[[1]](#footnote-2),[[2]](#footnote-3),[[3]](#footnote-4)


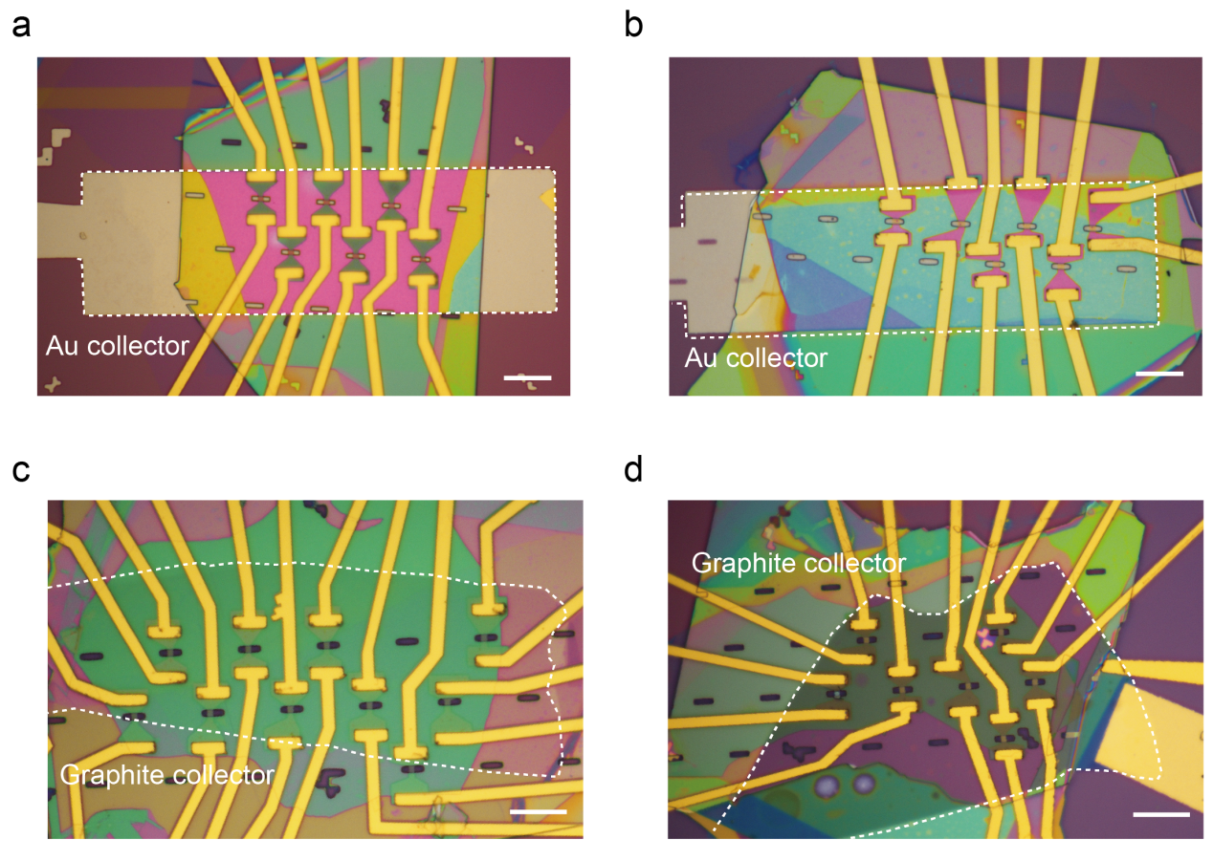


**Figure S3. Optical images of 2D vdW NOEMS.** (a)-(b) are devices with Au collectors. (c)-(d) are devices with graphite collectors, achieved using vdW stacking methods. Electrodes connecting sources, drains, and collectors are fabricated following the work flow described in Fig. 1 in the main text and Fig. S1. Scale bars are 10 μm.


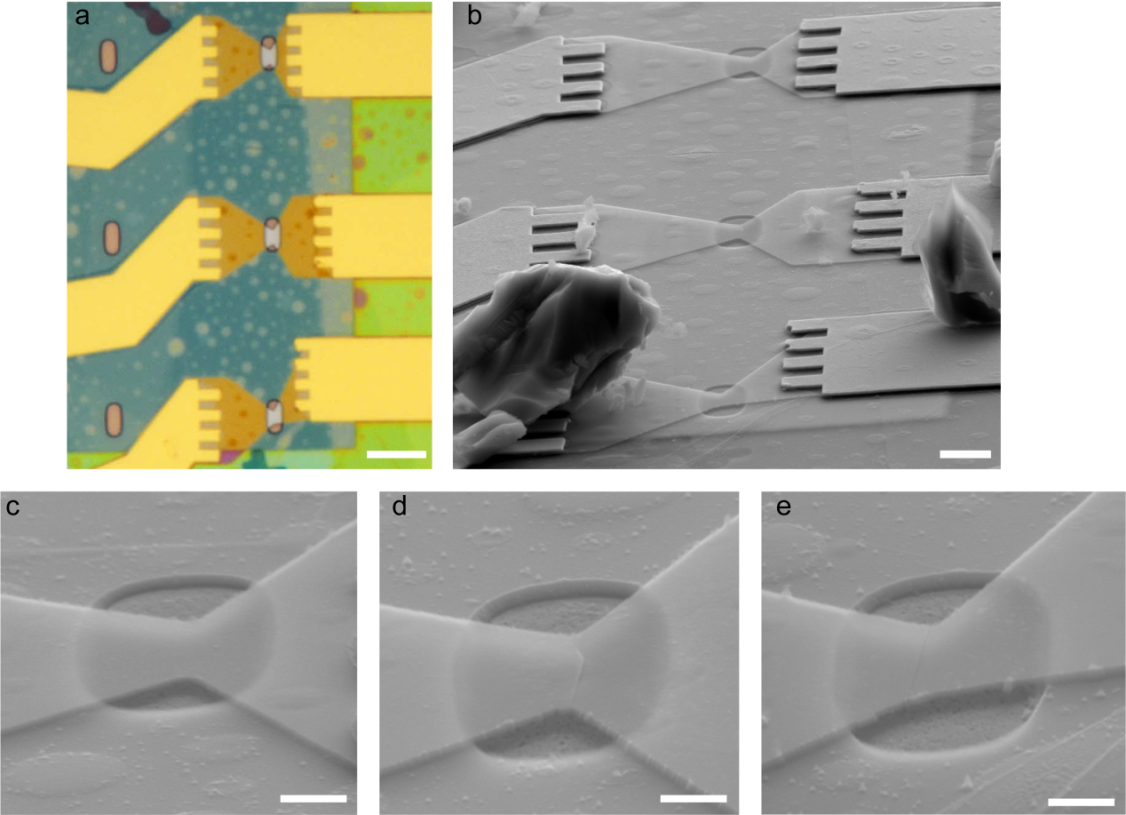


**Figure S4. vdW NOEMS serves as vertical thermionic emission diode with Au bottom collectors.** (a) Optical image of a typical device using monolayer graphene as the emission channel, scale bar is 10 μm. (b) Scanning electron micrograph (SEM) image of (a), scale bar is 2 μm. (c)-(e) are SEM images corresponding to the devices in (b) from top to bottom. Scale bars in (c)-(e) are 2 μm. Perfect suspension is shown in (c), and nano-cracks are visible in the suspended h-BN/graphene in (d) and (e), which are broken after several cycles of emission experiments. It is noticed that some samples suffer from cracks, while some others are collapsed after overloads of large current flowing, as also shown in Fig. S5.


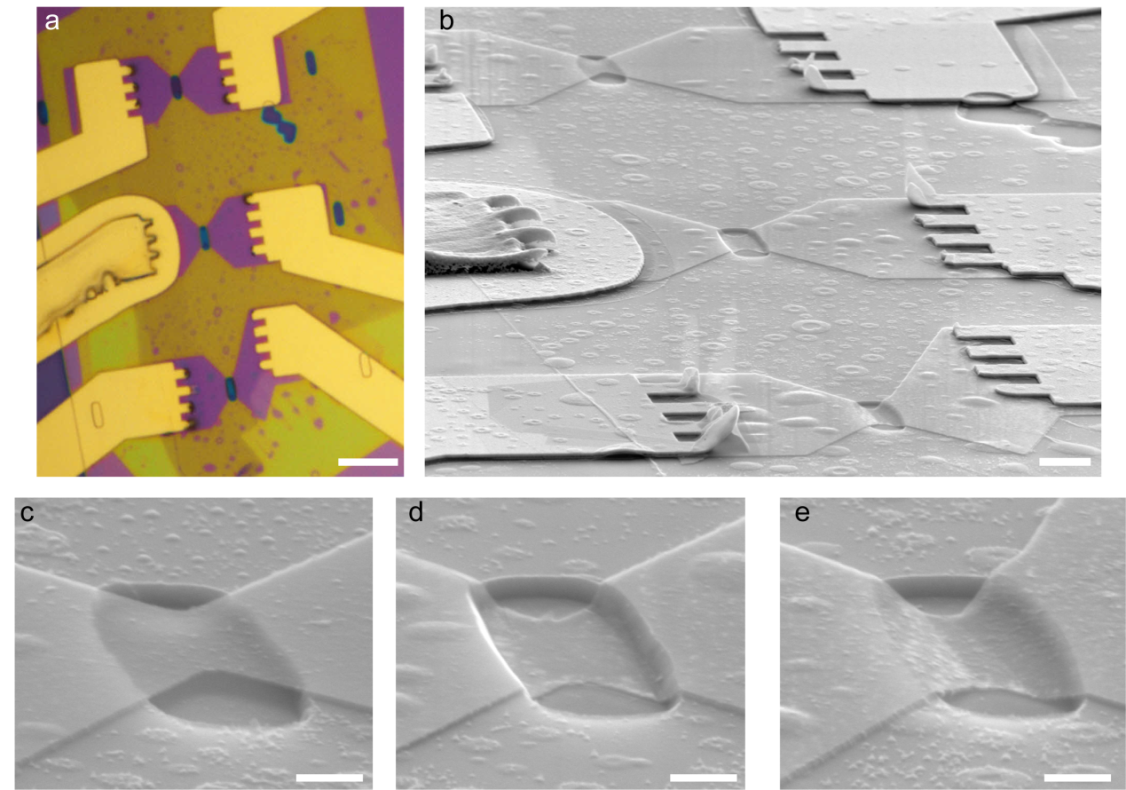


**Figure S5. vdW NOEMS serves as vertical thermionic emission diode with graphite bottom collectors.** (a) Optical image of a device using Graphene as the emission channel, and graphite as the collector. Scale bar is 10 μm. (b) SEM image of (a), scale bar is 2 μm. (c)-(e) are SEM images corresponding to (b) from top to bottom. Scale bars in (c)-(e) are 2 μm. The emitter channel in (d) collapsed during fabrication, while that in (e) collapsed after an overload of current in the thermionic electron emission measurements.


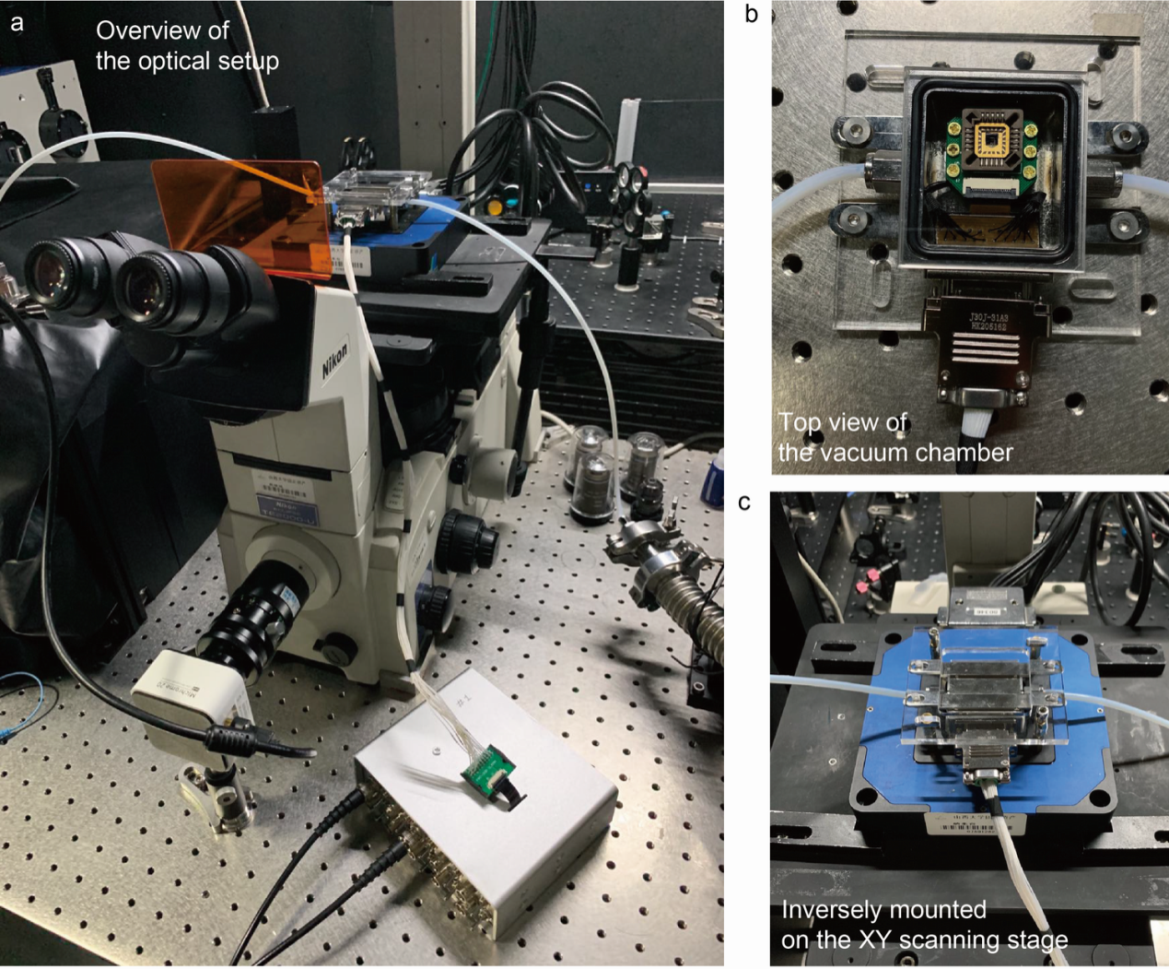


**Figure S6. Setup for simultaneous measurements of the optical spectrum and electrical transport in the vdW NOEMS.** (a) The overview of the setup. (b) Home-made vacuum chamber hosting a standard 20 pin plastic leaded chip carriers (PLCC) socket for the chip carrier, with the sample wire bonded and connected through a vacuum feed-through. A fused silica (JGS1) optical window was used as the seal plate. A 100 times objective with a working distance of 13 mm was used. (c) The whole chamber was mounted upside down on an X-Y positioner for mapping purposes.


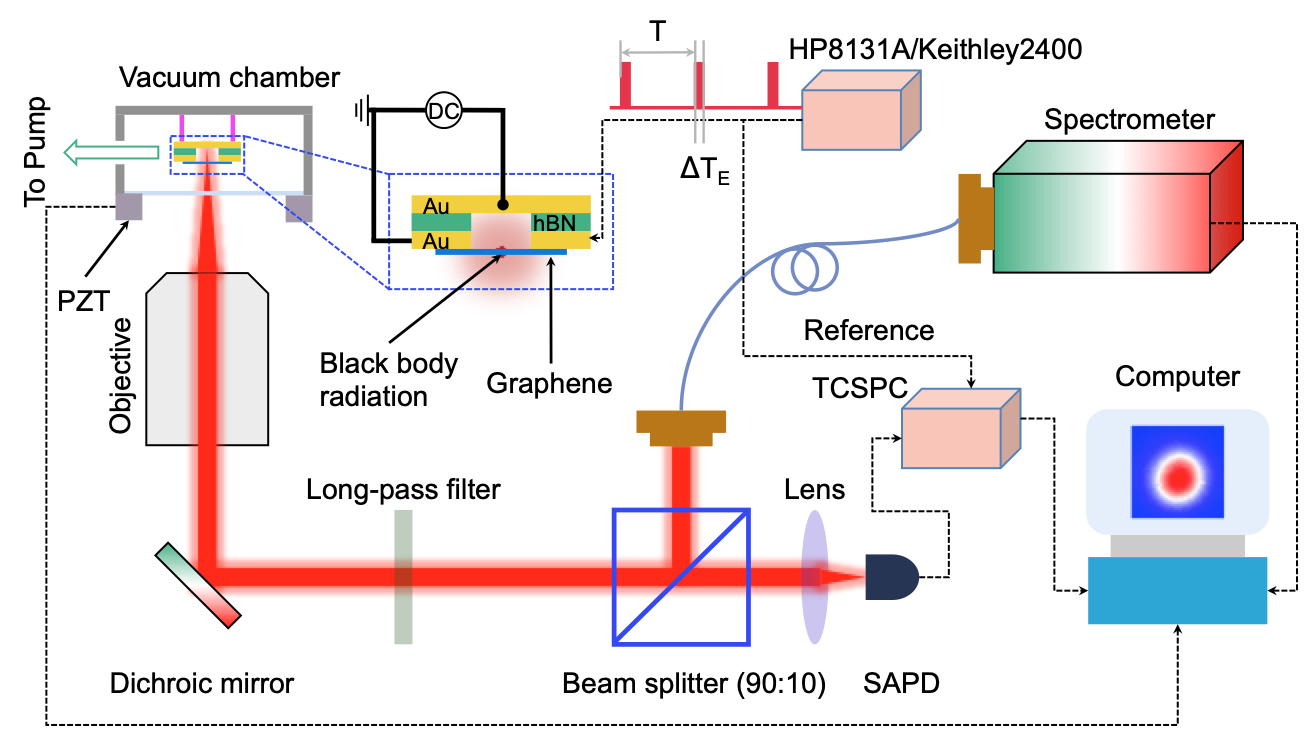


**Figure S7**. **Schematic diagram of the optical setup.** To perform optical measurements, a home-made vacuum chamber (4 cm×5 cm×2 cm) is used to hold the vertical vdW NOEMS under a vacuum of about 10^-2^ mbar. The vacuum chamber was inversely mounted on an X-Y scanning piezoelectric ceramic translation stage (PZT, Tritor, 200/20SG). The black body radiation (BBR) from graphene vacuum tubes was collected by an ultra-long working distance objective (Motic, 100×, NA=0.55, WD=13 mm). After passing a dichroic mirror (Semrock, Di03-R532-t1) and long-pass filter (Semrock, LP03-532RE-25), BBR was splitted by a beam splitter with the ratio of 90:10. The weaker part was focused by a lens and then detected by a single-photon avalanche diode (SPAD, PerkinElmer, SPCM-AQR-15), while the stronger part was transferred to a spectrometer (Andor, Shamrock, SR-303i) via an optical fiber. The spectroscopy of BBR was detected by a cooled charge-coupled device (CCD, iVac 316). For the static BBR, the DC voltage was applied by a Keithley 2400; while for the time-resolved BBR, a rectangular waveform of AC current was exerted into the sample by a HP8131A. The period of the AC voltage is *T*, with a pulse width of Δ*T*. The time-resolved spectra were recorded by a time-correlated single-photon counting (TCSPC) system with the temporal resolution about 64 ps. A reference voltage from the HP8131A was used as a trigger for the TCSPC.


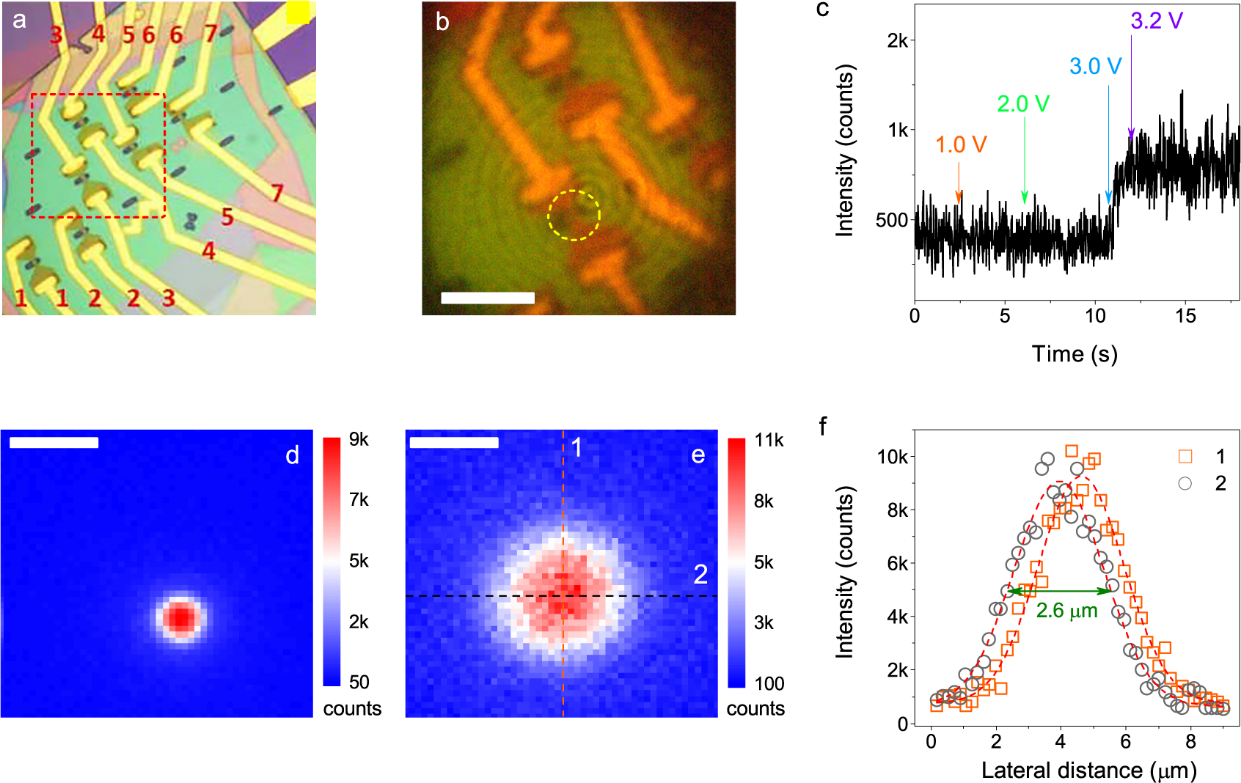


**Figure S8**. **Locating the center of thermionic emission samples and the measurements of black body radiation (BBR).** (a) Optical micrograph image of a vacuum tube sample (S-3). (b) Optical image of the test sample through an industrial camera mounted on the home-made optical system (Figs. S6-S7). The detection region has been highlighted by the dashed square in a. Scale bar: 10 μm. (c) The detected signal (via SPCM) during the tuning of *V*_ds_ (as the solid arrows shown in the figure). Mapping the BBR with a coarse step (d) and fine step (e), respectively. Scale bars in d and e are 10 μm and 2 μm, respectively. (f) The intensity profiles (circles and squares) along the dashed lines shown in e. The full width at half maximum was determined to be about 2.6 μm by a Gauss function. To perform the spectroscopy of the BBR of graphene vacuum tubes, we accurately positioned the center of the sample by an X-Y PZT scanning stage. Firstly, we located the region of the sample (pin 3-3, as the dashed circle shown in the figure) with an industrial camera and compared the picture with the optical micrograph image of the prepared sample, as shown in (a)-(b). The lateral resolution of this procedure is about 10 μm. Then, we applied a mild voltage of *V*_ds_ ~ 3 V (corresponds to an *I*_ds_ of ~ 10 mA) on the vacuum tube and monitored the BBR via SPCM. As shown in (c), a faint signal (~500 counts per second) indicated the BBR started to occur, which cannot be seen by an industrial camera. In this case, we mapped the full sample with a coarse step (600 nm per step) by PZT scanning stage to find the vacuum tube, as shown in (d). At last, the center position of the vacuum tube was located by mapping with a fine step (150 nm per step), as shown in (e). The positioning accuracy of our system is about 50 nm. To demonstrate the light-emitting region of the vacuum tube, we plotted the intensity profiles along the dashed lines shown in (f), which can be well fitted by a Gauss function. The light-emitting region was estimated to about 2.6 μm.


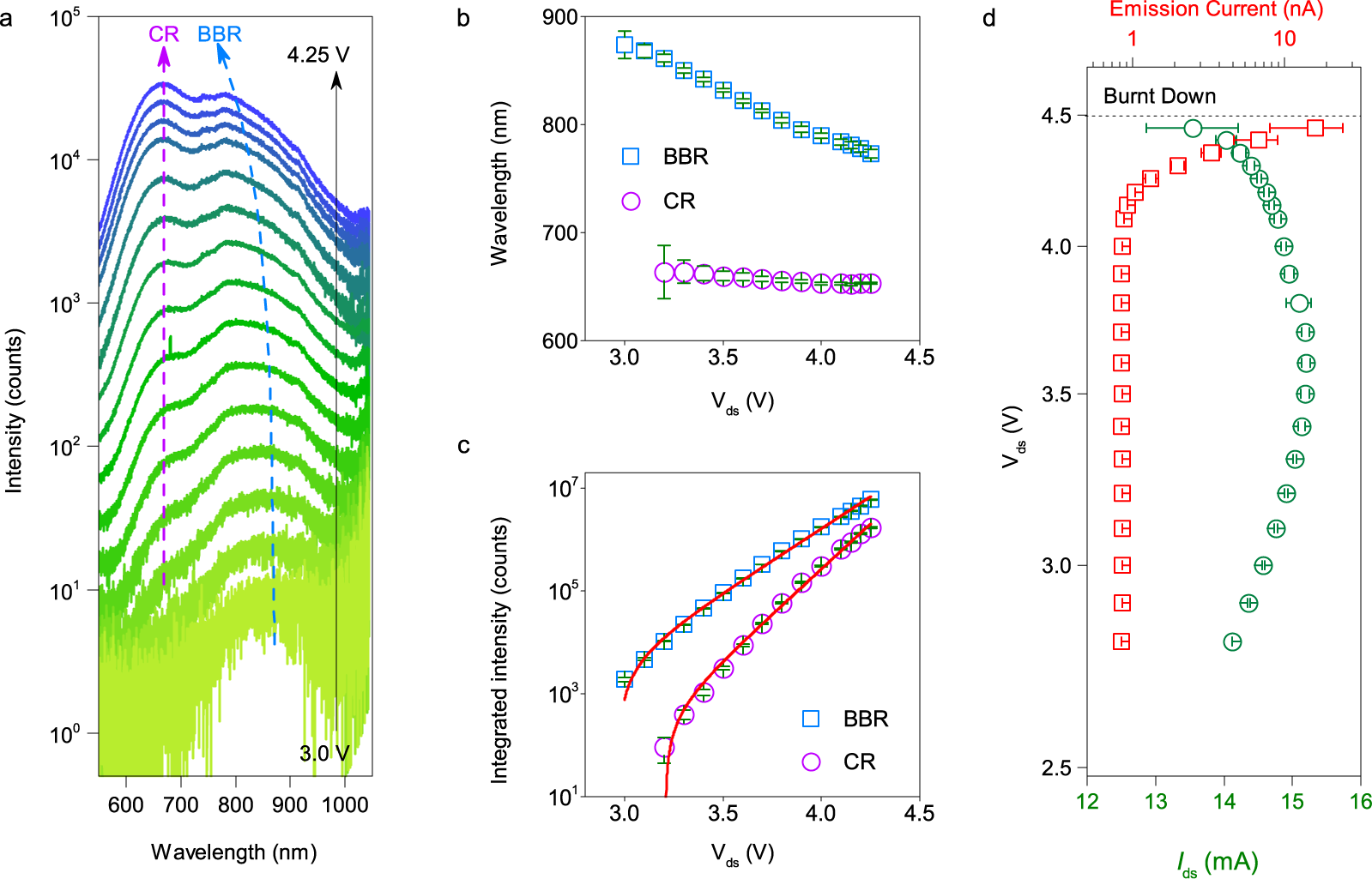


**Figure S9.** **Optical characterizations of the thermionic electron emission of a typical vdW NOEMS sample (Sample S-3, pin 7-7).** (a) BBR as a function of radiation wavelength at different *V*_ds_. The CR denotes the cavity resonance peaks. The center wavelengths of the two peaks and their integrated intensities as a function of *V*_ds_ are presented in b and c, respectively. The CR peak centers at about ~670 nm, while the BBR peak has a blue-shift from about 880 to 780 nm. The solid lines shown in c are the exponential fits. (d) Profiles of thermionic electron emission *I*_Emission_ (red squares) and *I*_ds_ (green circles) as a function of *V*_ds_. By fitting the central peak of BBR with the correction of detector efficiency, we obtained a temperature levitation from about 1700 K to 2750 K, with *V*_ds_ ranging from 3.0 V to 4.1 V. Interestingly, there is another peak in the optical spectra emerging at ~ 670 nm, and is enhanced with increased intensity of the central peak, even dominating the spectra at *V*_ds_ > 3.9 V. This behavior resembles the laser-like cavity resonance emission, which occurs when the cavity depth is a multiply of half the optical wavelength. In the tested sample, the depth of the cavity was obtained to be 322 nm, approximately half of the wavelength.


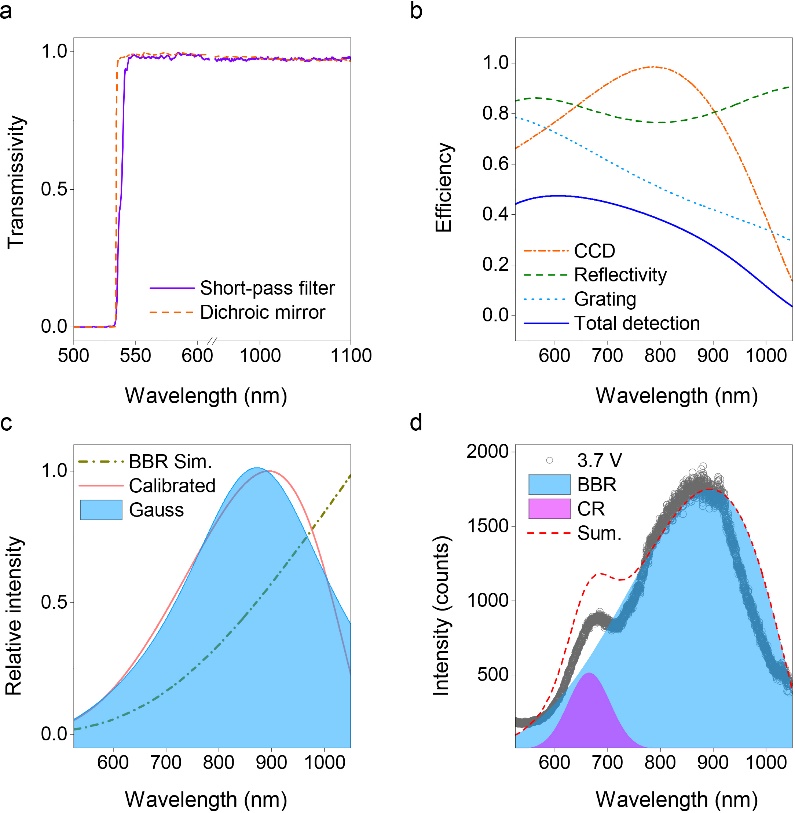


**Figure S10.** **Estimation of the black body radiation (BBR) temperature.** (a) The transmissivity of the long-pass filter and dichroic mirror. (b) Detection efficiency of our optical system as a function of wavelength. (c) Simulation and calibration of BBR. (d) Comparison of the experimental data and simulation curve.

Assuming the BBR from the graphene vacuum tubes is a quasi-equilibrium process, we can estimate the radiation temperature by the Planck radiation formula,

$I(\nu)\propto\nu^{3}\cdot\left[e^{\frac{h\nu}{kT}}-1\right]^{-1}$ (S1)

Prior to the estimation of BBR temperature, we first calibrated the detection efficiency of the optical system as a function of wavelength. Before detected by the CCD, BBR passed through the window glass of the vacuum chamber, the objective, the dichroic mirror, the long-pass filter, the beam splitter, the fiber, and then diffracted by the grating, and reflected by the mirror. Considering the transmissivity of the window glass, the objective, the optical fiber, and the reflectivity of the beam splitter are almost unity in the detection region (550 nm-1050 nm) of our optical system, thus the influences of these elements are ignored during our estimation. The transmissivity of the dichroic mirror and the long-pass filter are presented in Fig. S10a. Thus, the total detection efficiency of our optical system, *D*(ν), can be expressed as:

$D(\nu)=D_{CCD}(\nu)\times D_{Ref}(\nu)\times D_{Gra}(\nu)$ (S2)

Where *D_CCD_*(ν), *D_Ref_*(ν), *D_Gra_*(ν) represents the quantum yield of CCD, the reflection efficiency of optics coating, and the efficiency of the grating, respectively. Then, we simulated BBR through equation S1 at a given temperature (as the dash-dotted line shown in Fig. S10c with the temperature of 2875 K) and calibrated the curve by the expression:

$I'(\nu)=\frac{I(\nu)}{D(\nu)}$ (S3)

The calibrated BBR curves presented a peak (the solid line shown in Fig. S10c), which can be approximatively fitted by a Gauss function and demonstrated the center wavelength of the peak. At last, we deconvoluted the measured BBR spectroscopy by two Gauss functions (assigned to BBR and cavity resonance (CR)) and determined the center wavelength of BBR. By comparing the center wavelengths of the simulated curves and the experimental data, we can estimate the BBR temperature. The difference between the simulated curves and the experiment data possibly results from the un-calibrated detection efficiency of the other optical elements or the non-equilibrium emission of BBR.


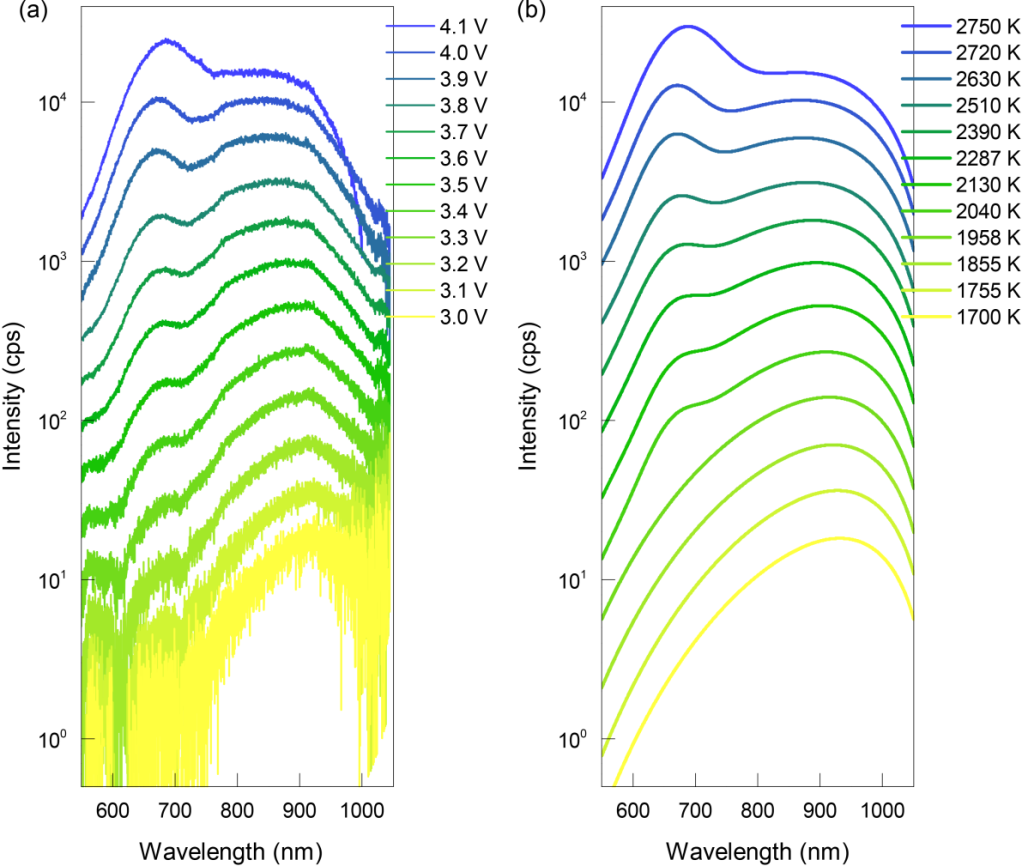


**Figure S11.** **Simulated emission spectra and the black body radiation temperatures**. (a) Black body radiation as a function of radiation wavelength at different *V*_ds_ (obtained in Sample S-3). (b) The simulated spectra by the method shown in Fig. S10, the temperature of black body radiation at each *V*_ds_ are indicated.


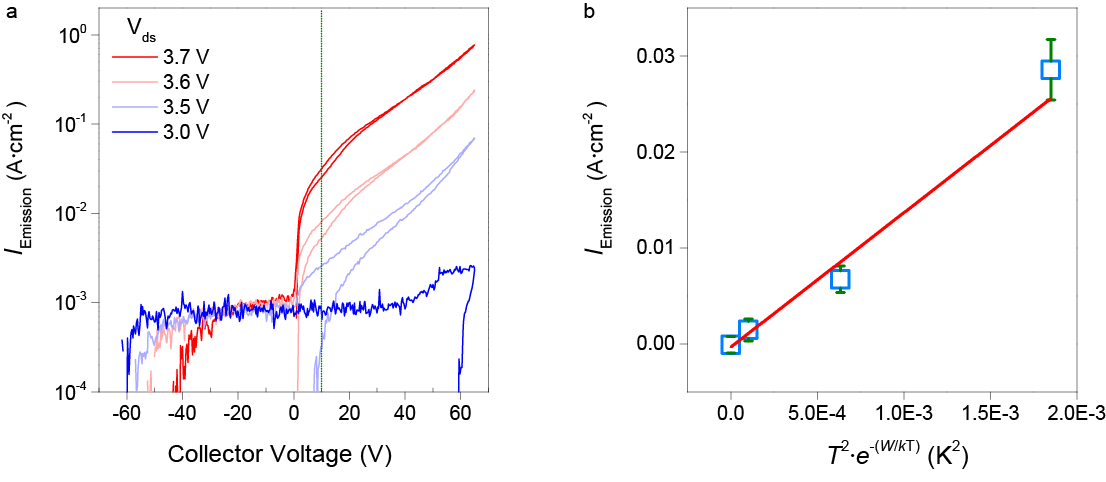


**Figure S12.** **Estimation of the Richardson constant.** (a) Thermionic emission current density *I*_Emission_ – *V*_collector_ curves at different *V*_ds_. The dot lines highlighted the collector voltage for the measurement of emission spectra. (Sample S-3) (b)The value of *T*^2^·exp(-*WK*^-1^*T^-1^*) as a function of *I*_Emission_, the solid line is the linear fit, with the slope to be 14.8 A·cm^-2^·K^-2^, and the linear correlation coefficients (R2) to be 0.953.


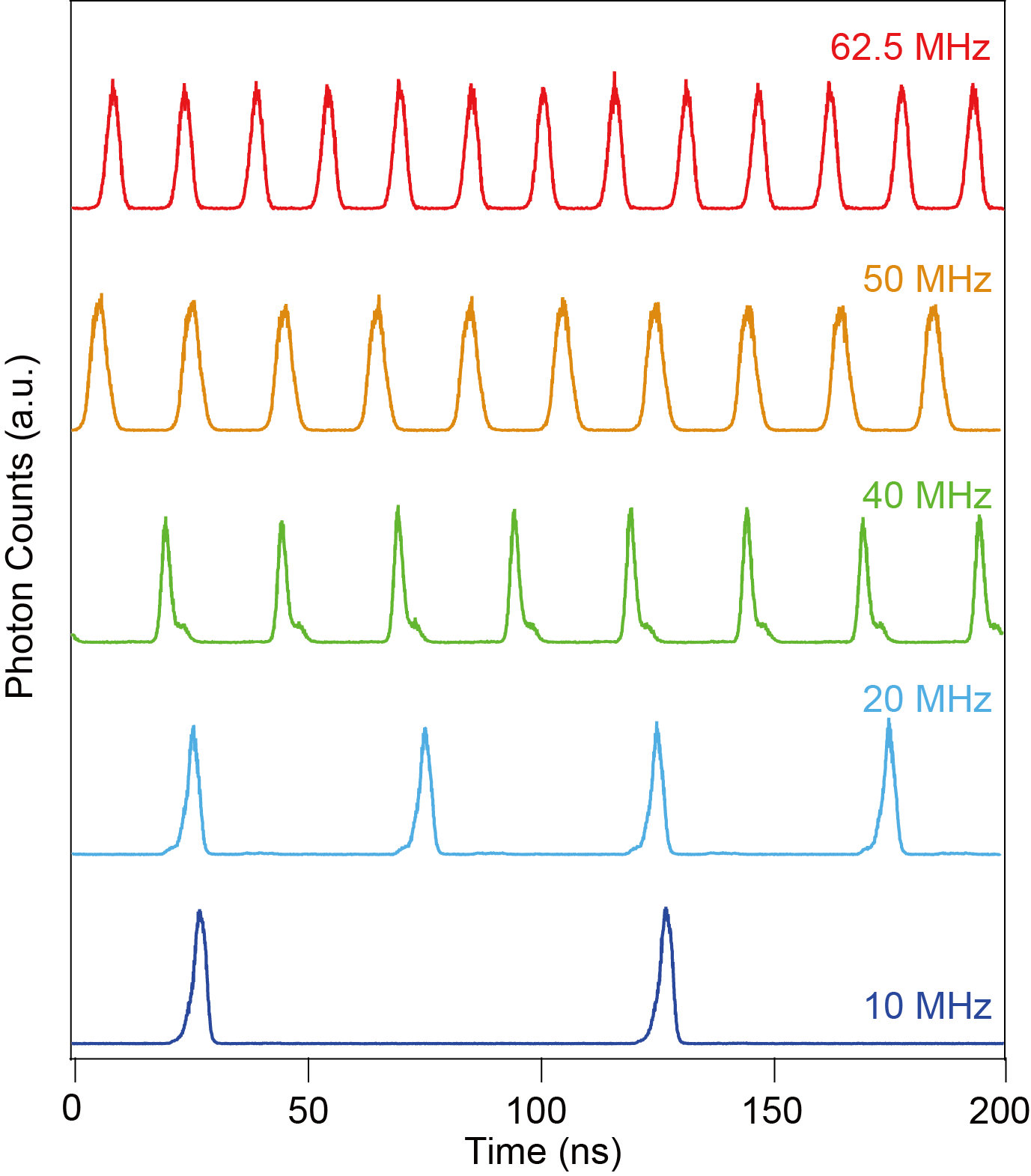


**Figure S13.** **Ultra-fast blackbody radiation in response to AC electrical current injection at different repetition frequencies.** The electrical pulse duration is kept at 10 ns, while the repetition rates are chosen to be 62.5 MHz, 50 MHz, 40 MHz, 20 MHz, and 10 MHz, respectively. A collection time scale of 200 ns is plotted. It is noticed that, when functioning as ns thermal radiators, the shortest pulse width performed in our devices is ~13 ns, which is comparable to other work,[[4]](#footnote-5) although being two orders wider than the fastest value to best of our knowledge,[[5]](#footnote-6) limited by the bandwidth of signal generator and cables of the measurement system.


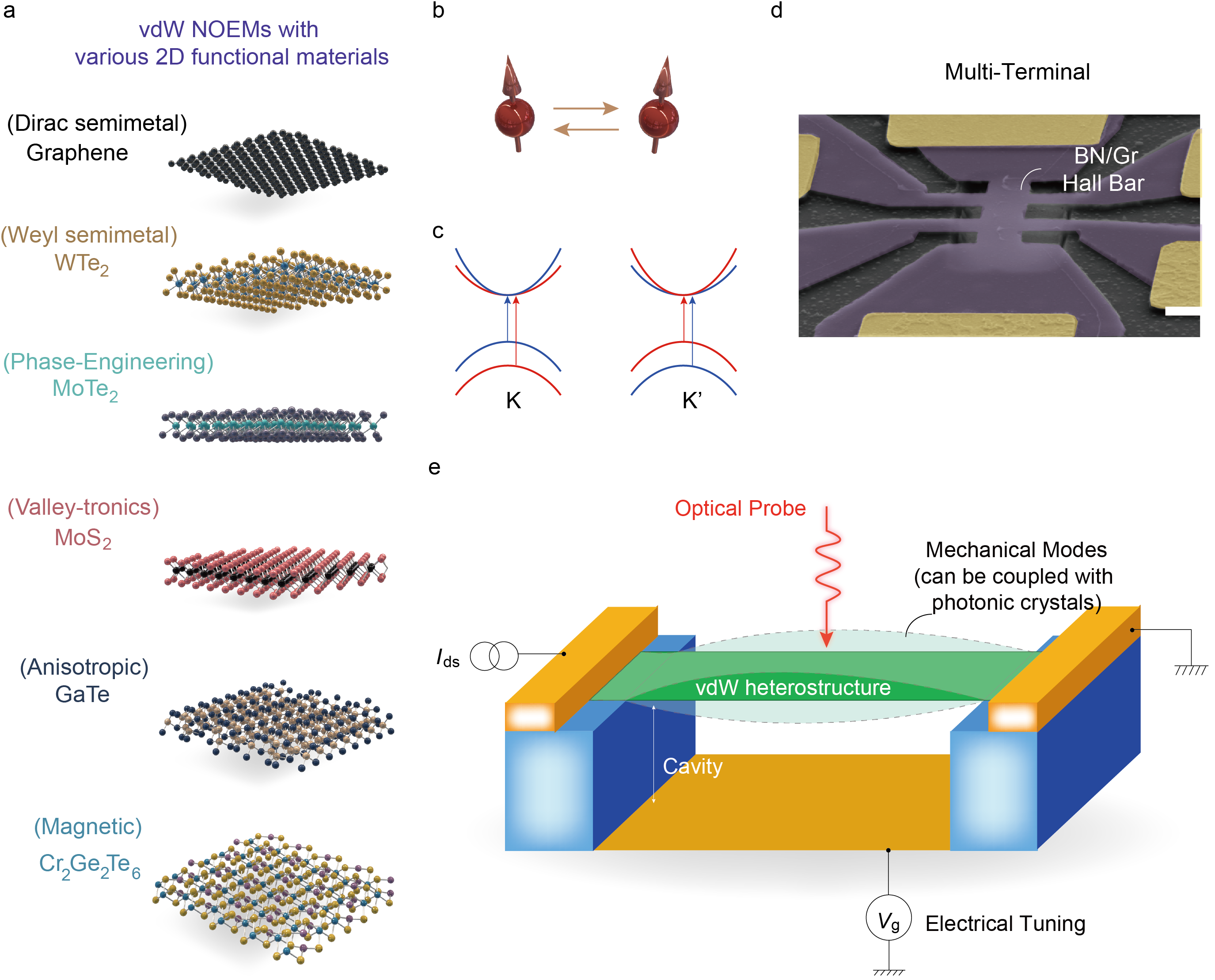


**Figure S14.** **Perspectives of the vdW heterostructure NOEMS.** (a) Schematic images of 2D materials with different functionalities. (b) and (c) Cartoon of the spin and valley interactions in 2D materials. (d) False-colored SEM image of a typical multi-terminal suspension of vdW NOEMS obtained using the method described in Fig.1 in the main text and Fig. S1. (e) Illustration of the NOEMS based on vdW heterostructures, which may be expanded into a broad range of 2D materials and their vertically stacked multilayers.


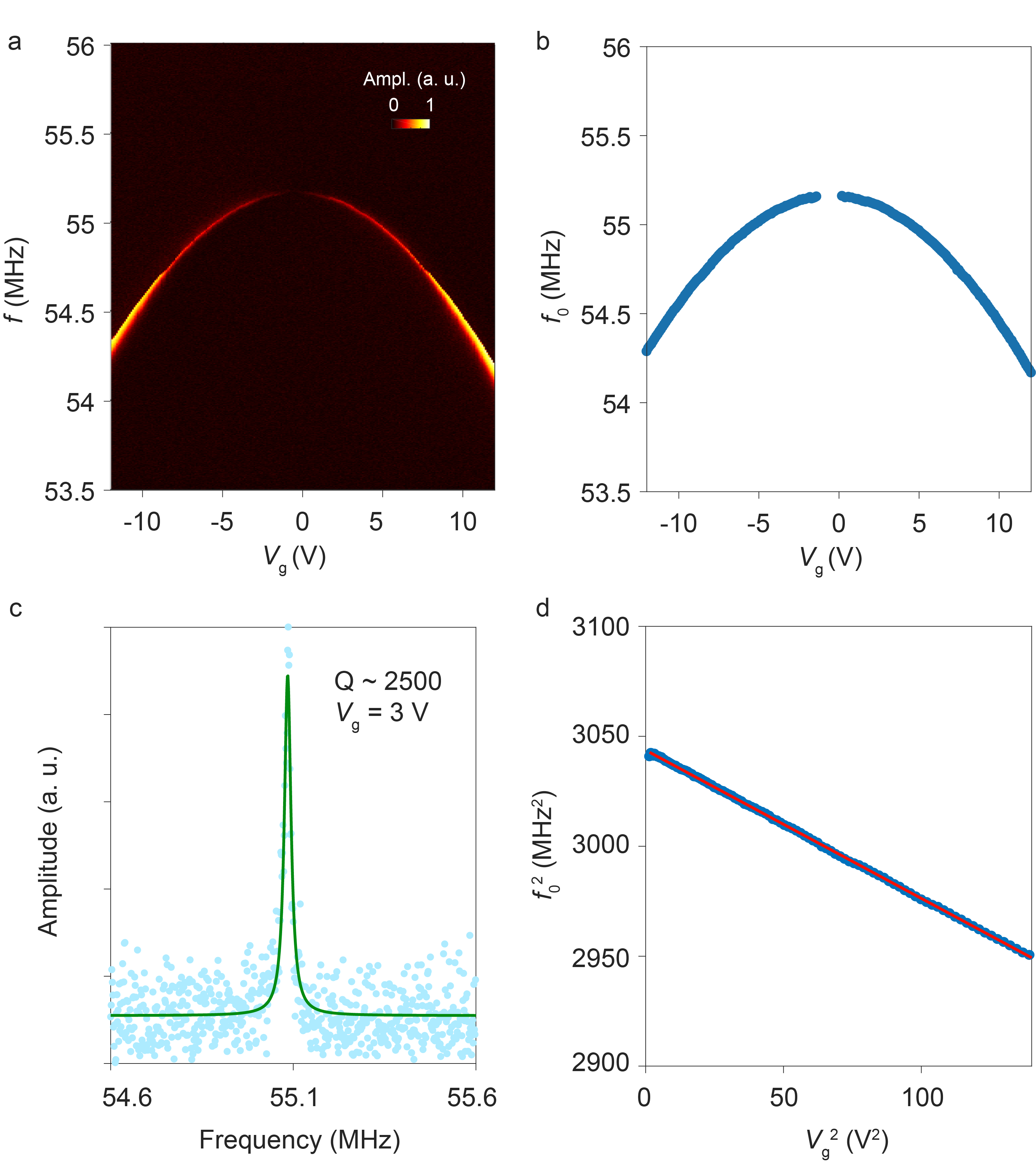


**Figure S15. Gate dependence of mechanical resonance of a CrOCl/graphene based NOEMS at 5 K, and 0 T.** (a) 2D mapping of resonance amplitude versus driven frequency and DC gate voltage *V*_g_. (b) Resonant frequency *f*_0_ extracted from (a) versus *V*_g_. (c) Typical mechanical resonance curve at *V*_g_ = 3 V. Dots are data and solid line is a Lorentzian fit with a Q-factor of ~ 2500. (d) Linear relation between *f*_0_^2^ and *V*_g_^2^. The red solid line is a linear fit of data (dots). Such linear relation reflects an effective spring constant *k*_eff_ ~ *V*_g_^2^, indicating large built-in strain of our devices.


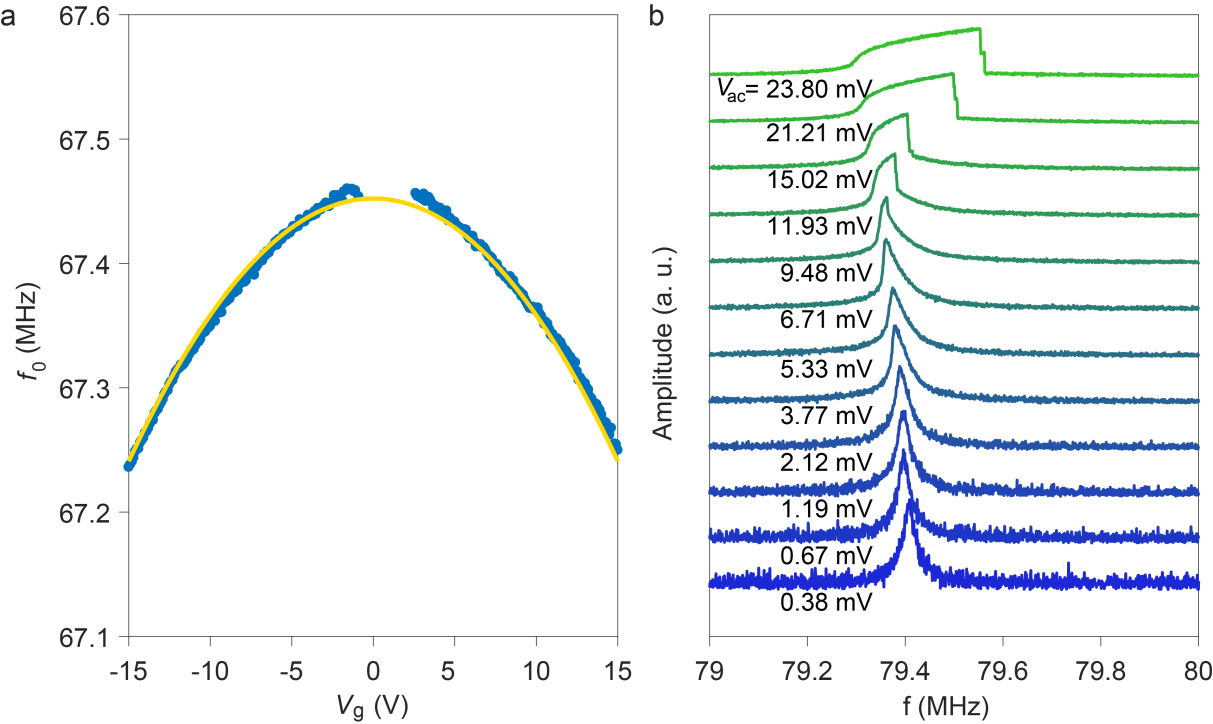


**Figure S16.** **Nonlinear mechanical response of a typical CrOCl based NOEMS.** (a) Resonant frequency *f*_0_ versus DC gate voltage *V*_g_ at an AC modulation voltage of *V*_ac_ ~ 2 mV. Solid line is a linear fit by using the method of Fig. S12d. For resonators with drum structure with sufficiently large built-in strain, we can estimate effective mass *m*_eff_ and initial strain ϵ from 4π*f*_0_^2^ = 4.92*Y*_eff_ ϵ*m*_eff_^-1^ – 0.271ε_0_π*R*^2^*V*_g_^2^(*m*_eff_·*z*_0_^3^)^-1^,[[[6]](#footnote-7),[[7]](#footnote-8)] where *Y*_eff_ ~ 2000 N/m is effective Young’s modulus, *R* = 1.5 μm is the radius of the drum, *z*_0_ = 300 nm is the distance from gate to the membrane. *m*_eff_ and ϵ are calculated to 1.3×10^-16^ kg and 0.48%, respectively. (b) Mechanical resonance curves at different *V*_ac_. *V*_g_ is set to 10 V. Each curve is vertically shifted for clarity. For *V*_ac_ larger than ~ 2 mV, obvious nonlinear mechanical response of the resonator can be seen, which is related to a critical vibration amplitude *z*_critical_ of ~ 2 nm. Ignoring higher order nonlinearity, we obtain the effective Duffing constant α_eff_ from an simplified equation α_eff_ = 1.54 *m*_eff_ (2π*f*_0_) 2/(Q *z*_critical_^2^), where Q ~ 2200 is the quality factor of the NOEMS. From the above mentioned parameters, the effective Duffing nonlinearity of the CrOCl based NOEMS is estimated to ~ 4.0×10^15^.


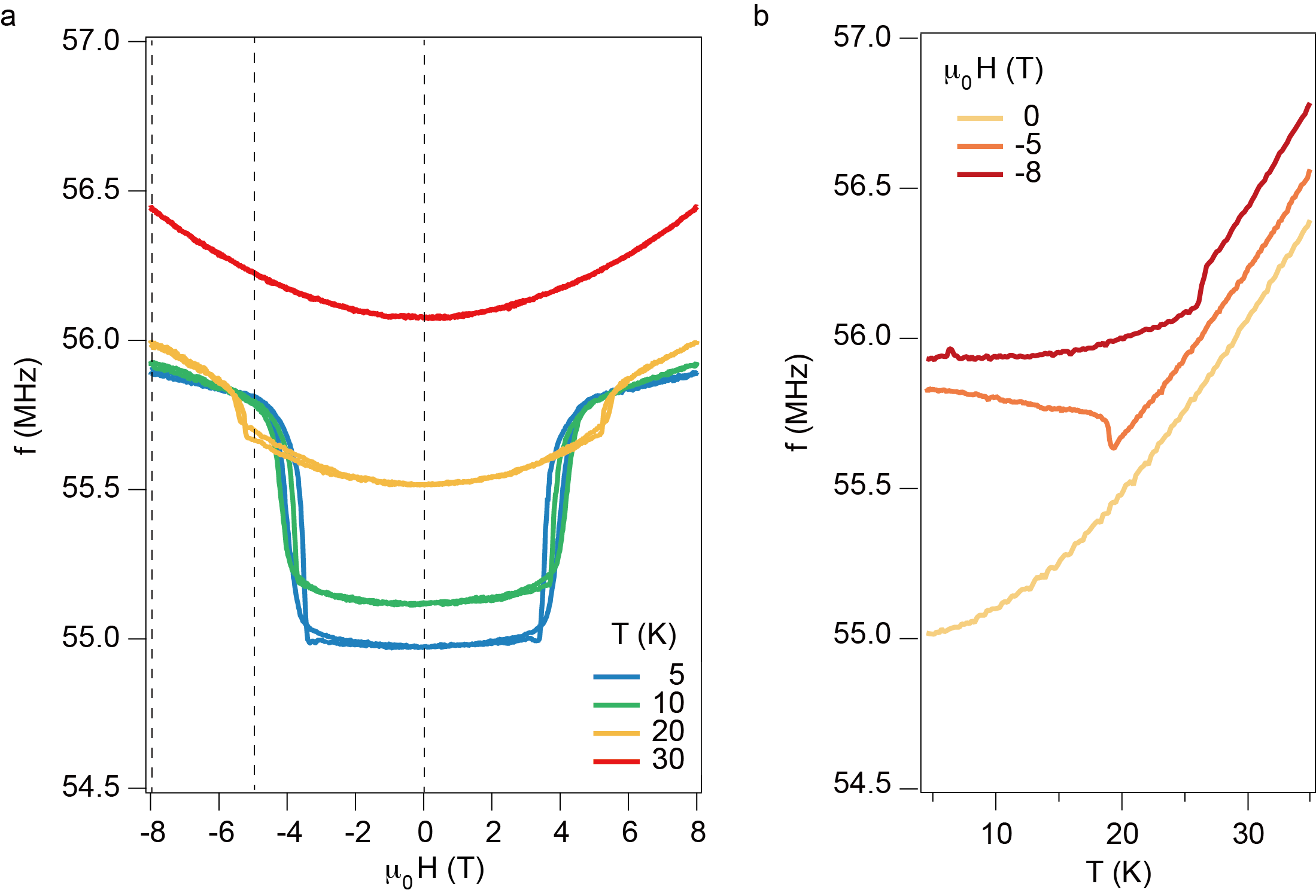


**Figure S17.** **Temperature dependence of the magnetic phase transition in a CrOCl/graphene NOEMS.** (a) Resonance frequency *f*_0_ as a function of perpendicular magnetic field, at different temperatures. (b) Line profiles of *f*_0_ versus temperature, obtained at different magnetic fields.

**
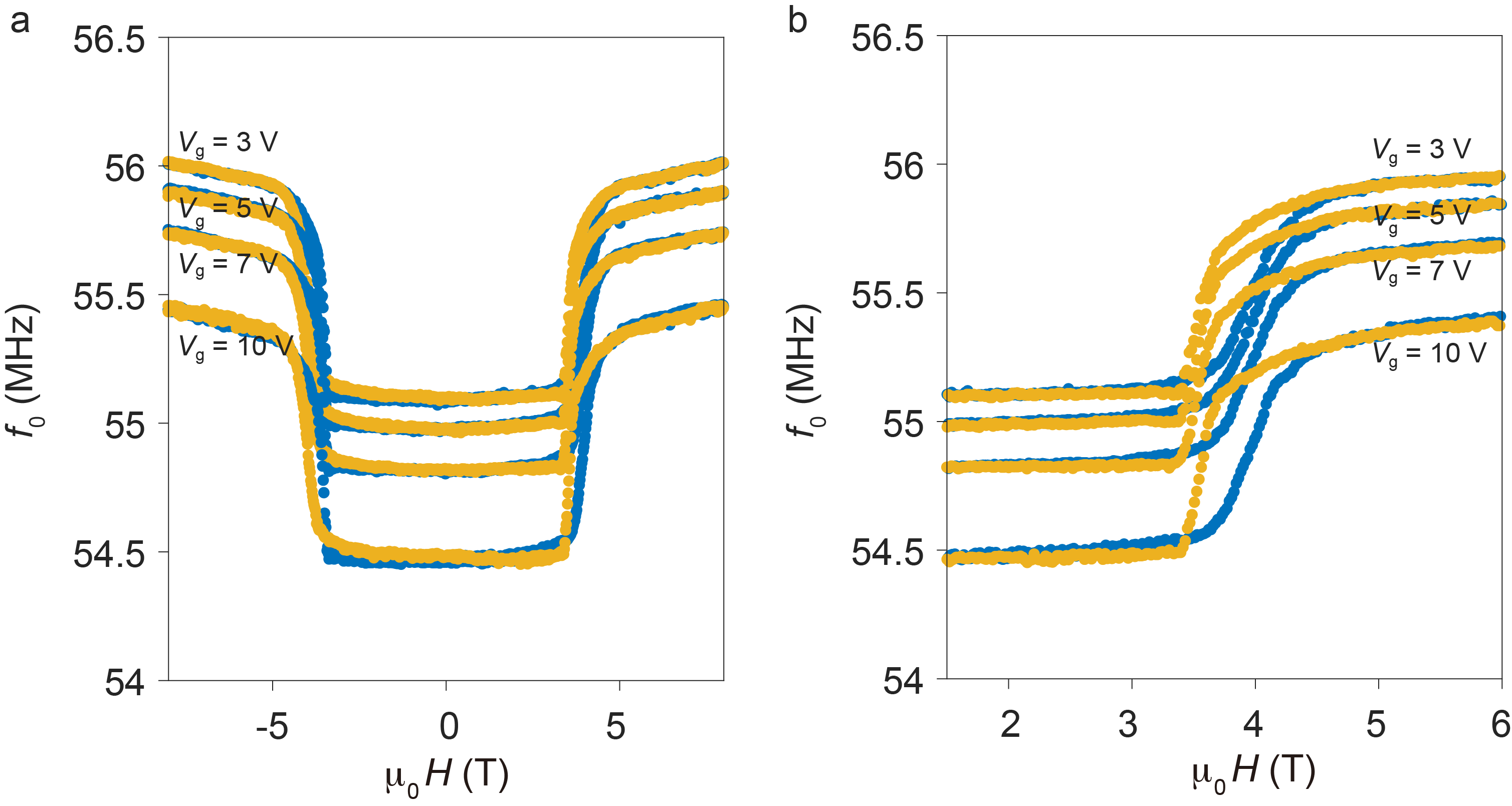
**

**Figure S18. Magnetic field dependence of mechanical resonant frequency of a CrOCl based NOEMS at typical DC *V*_g_.** (a) *f*_0_ – *H* curves at *V*_g_ = 3 V, 5 V, 7 V, and 10 V, respectively. (b) *f*_0_ – *H* curves in a range of 1.5 T to 6 T at several typical DC gate voltages. Within our measurement uncertainty, the magnetic transition fields of the CrOCl NOEMS are unchanged for those gate voltages. Data obtained at 5 K.

**Table S1. Summarization of characteristic parameters nanosized thermionic emission diodes.**

| Reference | On/Off emission ratio | Collector Voltage (V) | On state *V*_c_ (V) | *I*_on_ per Channel | SS (mV/decade) |
| --- | --- | --- | --- | --- | --- |
| This work | 10^5^ | <1 | <1 | 20 nA | ~200 |
| [[[8]](#footnote-9)] | 10^6^ | 15 | 4.5 | 10 nA | 120 |
| [[[9]](#footnote-10)] | 10^6^ | 100 | N/A | 10 nA | N/A |
| [[[10]](#footnote-11)] | 10^3^ | 210 | N/A | N/A | 158 |
| [[[11]](#footnote-12)] | 10^6^ | 6 | 1 | N/A | N/A |
| [[[12]](#footnote-13)] | N/A | 40 | 20 | 170 nA | N/A |

1. Singh, V., Sengupta, S., Solanki, H. S., Dhall, R., Allain, A., Dhara, S., Pant, P., and Deshmukh, M. M. Probing thermal expansion of graphene and modal dispersion at low-temperature using graphene nanoelectromechanical systems resonators. Nanotechnology **21**,165204 (2010). [↑](#footnote-ref-2)
2. Manzeli, S., Dumcenco, D., Marega, M. M. &Kis, A. Self-sensing, tunable monolayer MoS_2_ nanoelectromechanical resonators. Nature Communications **10**, 4831 (2019). [↑](#footnote-ref-3)
3. Jiang, S., Xie, H., Shan, J.  and Mak, J. F. Exchange magnetostriction in two-dimensional antiferromagnets. Nature Materials **19**, 1295–1299 (2020). [↑](#footnote-ref-4)
4. Wu, G., Wei, X., Gao, S., Chen, Q. & Peng, L. Tunable graphene micro-emitters with fast temporal response and controllable electron emission. Nature Communications **7**, 11513 (2016). [↑](#footnote-ref-5)
5. Kim, Y. D. *et al.* Ultrafast Graphene Light Emitters. Nano Lett **18**, 934−940 (2018). [↑](#footnote-ref-6)
6. V. A. Sazonova, A tunable carbon nanotube resonator. PhD thesis, 2006. [↑](#footnote-ref-7)
7. C. Chen, Graphene NanoElectroMechanical Resonators and Oscillators. PhD thesis, 2013. [↑](#footnote-ref-8)
8. Wu, G., Wei, X., Zhang, Z., Chen, Q. & Peng, L. Agraphene-based vacuum transistor with a high on/off current ratio. Advanced Functional Materials 25, 59725978(2015). [↑](#footnote-ref-9)
9. Wu, G., Wei, X., Gao, S., Chen, Q. & Peng, L. Tunable graphene micro-emitters with fast temporal response and controllable electron emission. Nature Communications 7, 11513 (2016). [↑](#footnote-ref-10)
10. Wang, Y. et al. High-performance on-chip thermionic electron micro-emitter arrays based on super-aligned carbon nanotube films. Advanced Functional Materials 30,1907814 (2020). [↑](#footnote-ref-11)
11. Murakami, H., Hirakawa, M., Tanaka, C. &Yamakawa, H. Field emission from well-aligned, patterned, carbon nanotube emitters. Applied Physics Letters 76, 1776(2000). [↑](#footnote-ref-12)
12. Lee, S. W. & Lee, E. H., S. S. & Yang. A study on field emission characteristics of planar graphene layers obtained from a highly oriented pyrolyzed graphite block. Nanoscale Res Lett 4, 1218–1221 (2009). [↑](#footnote-ref-13)
